# Engineering the kinetic stability of a β-trefoil protein by tuning its topological complexity

**DOI:** 10.3389/fmolb.2023.1021733

**Published:** 2023-02-08

**Authors:** Delaney M. Anderson, Lakshmi P. Jayanthi, Shachi Gosavi, Elizabeth M. Meiering

**Affiliations:** ^1^ Department of Chemistry, University of Waterloo, Waterloo, ON, Canada; ^2^ Simons Centre for the Study of Living Machines, National Centre for Biological Sciences, Tata Institute of Fundamental Research, Bangalore, India

**Keywords:** protein engineering, kinetic stability, protein topology, structure-based models, β-trefoil, long-range order, absolute contact order

## Abstract

Kinetic stability, defined as the rate of protein unfolding, is central to determining the functional lifetime of proteins, both in nature and in wide-ranging medical and biotechnological applications. Further, high kinetic stability is generally correlated with high resistance against chemical and thermal denaturation, as well as proteolytic degradation. Despite its significance, specific mechanisms governing kinetic stability remain largely unknown, and few studies address the rational design of kinetic stability. Here, we describe a method for designing protein kinetic stability that uses protein long-range order, absolute contact order, and simulated free energy barriers of unfolding to quantitatively analyze and predict unfolding kinetics. We analyze two β-trefoil proteins: hisactophilin, a quasi-three-fold symmetric natural protein with moderate stability, and ThreeFoil, a designed three-fold symmetric protein with extremely high kinetic stability. The quantitative analysis identifies marked differences in long-range interactions across the protein hydrophobic cores that partially account for the differences in kinetic stability. Swapping the core interactions of ThreeFoil into hisactophilin increases kinetic stability with close agreement between predicted and experimentally measured unfolding rates. These results demonstrate the predictive power of readily applied measures of protein topology for altering kinetic stability and recommend core engineering as a tractable target for rationally designing kinetic stability that may be widely applicable.

## 1 Introduction

Proteins are increasingly used in industrial, medical, and research applications, and demand for industrially useful proteins is growing worldwide. However, most proteins exhibit only moderate stability, making them ill-suited to the harsh conditions routinely used in biotechnical applications (Magliery, 2015). As such, improving protein stability is a primary focus in many protein engineering endeavors (Bornscheuer et al., 2012; Sun et al., 2019; Teufl et al., 2022).

Protein stability is a complex interplay between kinetic and thermodynamic stabilities. Kinetic stability is determined by the free energy barrier of unfolding between a protein’s native state and transition state (Sanchez-Ruiz, 2010; Sun et al., 2019). Kinetically stable proteins have high activation free energy barriers and unfold slowly (Broom et al., 2015b). In contrast, thermodynamic stability refers to the free energy difference between a protein’s native and unfolded states (Nisthal et al., 2019). Thermodynamically stable proteins have lower Gibbs free energy in their folded state relative to their unfolded state, and their equilibrium favors the folded protein (Sanchez-Ruiz, 2010). So, kinetic stability dictates a protein’s folded, functional lifetime, while thermodynamic stability determines the population of native protein at equilibrium. Proteins can exert their biological function with low thermodynamic stability so long as they have sufficient kinetic stability to maintain their folded state over an adequate physiological timescale (Sanchez-Ruiz, 2010). Notably, increased kinetic stability correlates with enhanced resistance to proteolytic degradation and aggregation (Broom et al., 2015b; Colón et al., 2017), longer shelf life (Luo et al., 2002), improved catalytic yield (McLendon and Radany, 1978), and higher tolerance to thermal and chemical denaturing conditions (Brissos et al., 2014; Broom et al., 2015b). Thus, kinetic stability is crucial in determining protein stability for practical applications.

To date, few studies address the rational design of kinetic stability. Previous strategies for improving protein kinetic stability focus on increasing protein rigidity through the introduction of disulfide bonds and proline residues (Clarke and Fersht, 1993; Mansfeld et al., 1997; Van den Burg et al., 1999; Pikkemaat et al., 2002; Liu et al., 2021) or by mutating residues with high crystallographic B-factors (Le et al., 2012; Xie et al., 2014; Chen et al., 2015; Duan et al., 2016; Liu et al., 2021). Recently, high-temperature Molecular Dynamics (MD) simulations have been used to target thermally flexible residues in a fashion similar to B-factor engineering with reasonable success (Pikkemaat et al., 2002; Xie et al., 2014; Quezada et al., 2018; Liu et al., 2021). However, prioritizing protein rigidity is not ideal for all protein applications, since rigid proteins tend to display diminished catalytic activity due to reduced conformational dynamics (Kim et al., 2012; Xie et al., 2014). Additionally, these methods are often labor-intensive, their experimental outcomes are difficult to predict, and the mutants studied show no unified molecular mechanism for kinetic stabilization. So, despite variable success, molecular determinants of kinetic stability remain poorly understood and no reliable approach for engineering kinetic stability is established (Sanchez-Ruiz, 2010; Musil et al., 2019; Sun et al., 2019).

Large-scale kinetic analyses and protein folding simulations using structure-based models coarse-grained to a C_α_ bead (C_α_-SBM) suggest that protein topology as measured by long-range order (LRO) and absolute contact order (ACO) may be useful for predicting protein kinetic stability (Gosavi, 2013; Broom et al., 2015a; 2015b). LRO reports structural complexity based on the number of long-range contacts normalized to protein chain length (Gromiha and Selvaraj, 2001), while ACO reflects the relative importance of local and non-local contacts in the native protein (Ivankov et al., 2003). Increasing LRO and ACO correlate strongly with decreasing protein unfolding rates at the transition midpoint (Broom et al., 2015a). Critically, comparing protein stabilities analyzed using a linear free energy extrapolation model under conditions of equal thermodynamic stability at the transition midpoint, i.e., where the equilibrium free energy of unfolding ( ${\Delta G}_{u}$ ) is zero, allows identification of differences in kinetic unfolding barrier heights (Gosavi, 2013; Broom et al., 2015a; 2015b). In a separate study, Broom et al. (2015b) found that free energy barriers simulated using C_α_-SBMs increase with increasing LRO/ACO and showed that proteins with high LRO/ACO tend to have longer half-lives. Additionally, Chavez et al. (2004) found that unfolding free energy barrier heights simulated using C_α_-SBM correlate well with protein unfolding rates at the protein folding temperature (T_f_), the simulation temperature equivalent to the transition midpoint. Thus, LRO, ACO, and C_α_-SBM simulations may provide valuable predictive tools for designing protein kinetic stability.

**FIGURE 1:**
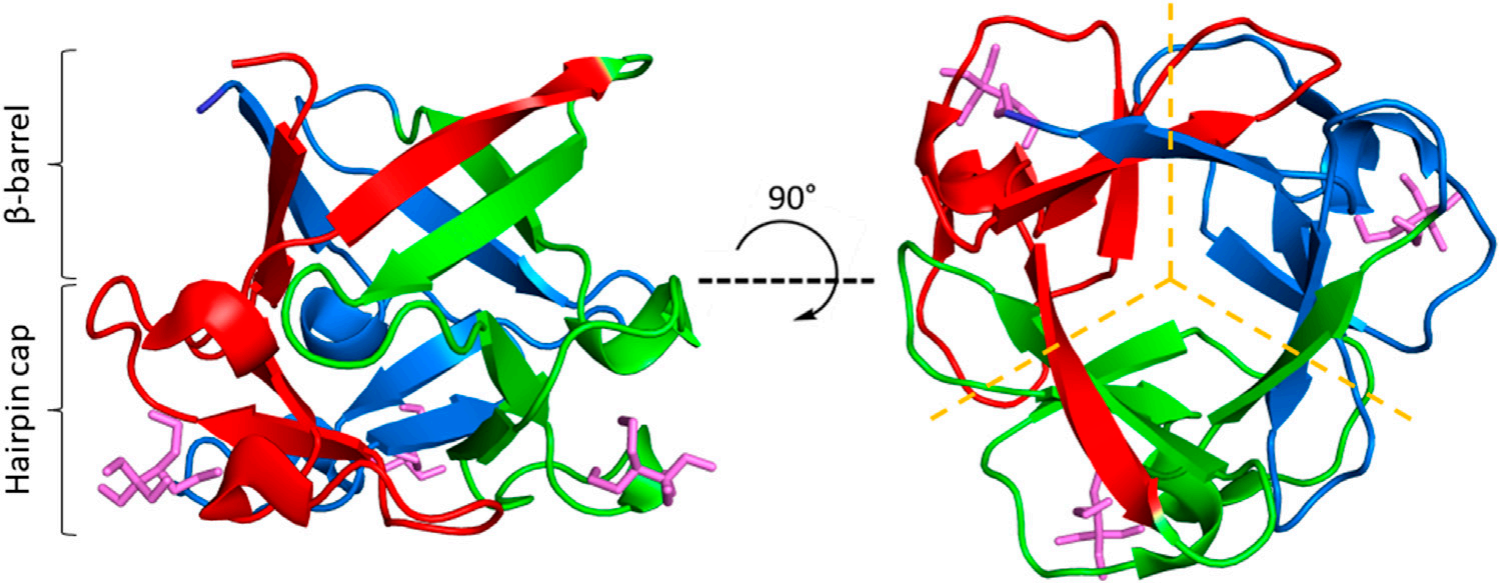
The β-trefoil fold. ThreeFoil (PDB ID: 3PG0) is a representative of the β-trefoil fold. β-trefoils consist of 12 β-strands arranged in a β-barrel and a β-hairpin cap (left). β-strands are connected by loops and turns of variable length. β-trefoils bind diverse ligands, often through their loops (ligands shown in purple). β-trefoils display internal pseudo three-fold symmetry (right). Symmetric trefoils are indicated by dashed yellow lines.

β-trefoil proteins offer a compelling model for using LRO, ACO, and C_α_-SBM unfolding free energy barriers to guide kinetic stability design. β-trefoils are comprised of 12 β-strands arranged into a six-stranded β-barrel and a six-stranded triangular cap, where one hairpin from the barrel and one from the cap form each trefoil (Figure 1) (Murzin et al., 1992; Broom et al., 2012). Alternating residues in each β-strand point inward to form the protein core, which consists of 18 highly conserved hydrophobic residues (Murzin et al., 1992). Despite their common fold, β-trefoils display great diversity in primary sequences and a wide range of stabilities (Murzin et al., 1992; Ponting and Russell, 2000; Brych et al., 2001; Broom et al., 2012; Gosavi, 2013). Of particular interest are the β-trefoil proteins ThreeFoil (3Foil) and hisactophilin (wtHis). 3Foil is a 3-fold symmetric designed protein that displays extreme kinetic stability with an unfolding half-life of ∼8 years (Broom et al., 2012; Broom et al., 2015b). Previous C_α_-SBM simulations showed that 3Foil’s remarkable kinetic stability arises from numerous long-range contacts between loop residues and across the protein core, which result in an unusually large unfolding free energy barrier (Broom et al., 2015b). In contrast, the natural β-trefoil protein wtHis has markedly fewer long-range contacts, a low unfolding barrier (Gosavi, 2013; Broom et al., 2015b), and moderate kinetic stability with a typical unfolding half-life of minutes to hours (Smith et al., 2010). Notably, core residues in wtHis form a functional cavity that spans the protein core (Smith et al., 2010; Shental-Bechor et al., 2012; MacKenzie et al., 2022). This cavity precludes the formation of long-range contacts between core residues and contributes to wtHis’ low LRO and unfolding free energy barrier. Equivalent residues in 3Foil are tightly packed and form many stabilizing long-range intramolecular contacts that contribute significantly to 3Foil’s high LRO. These core residues offer a clear starting point for modulating kinetic stability in wtHis and 3Foil.

**FIGURE 2:**
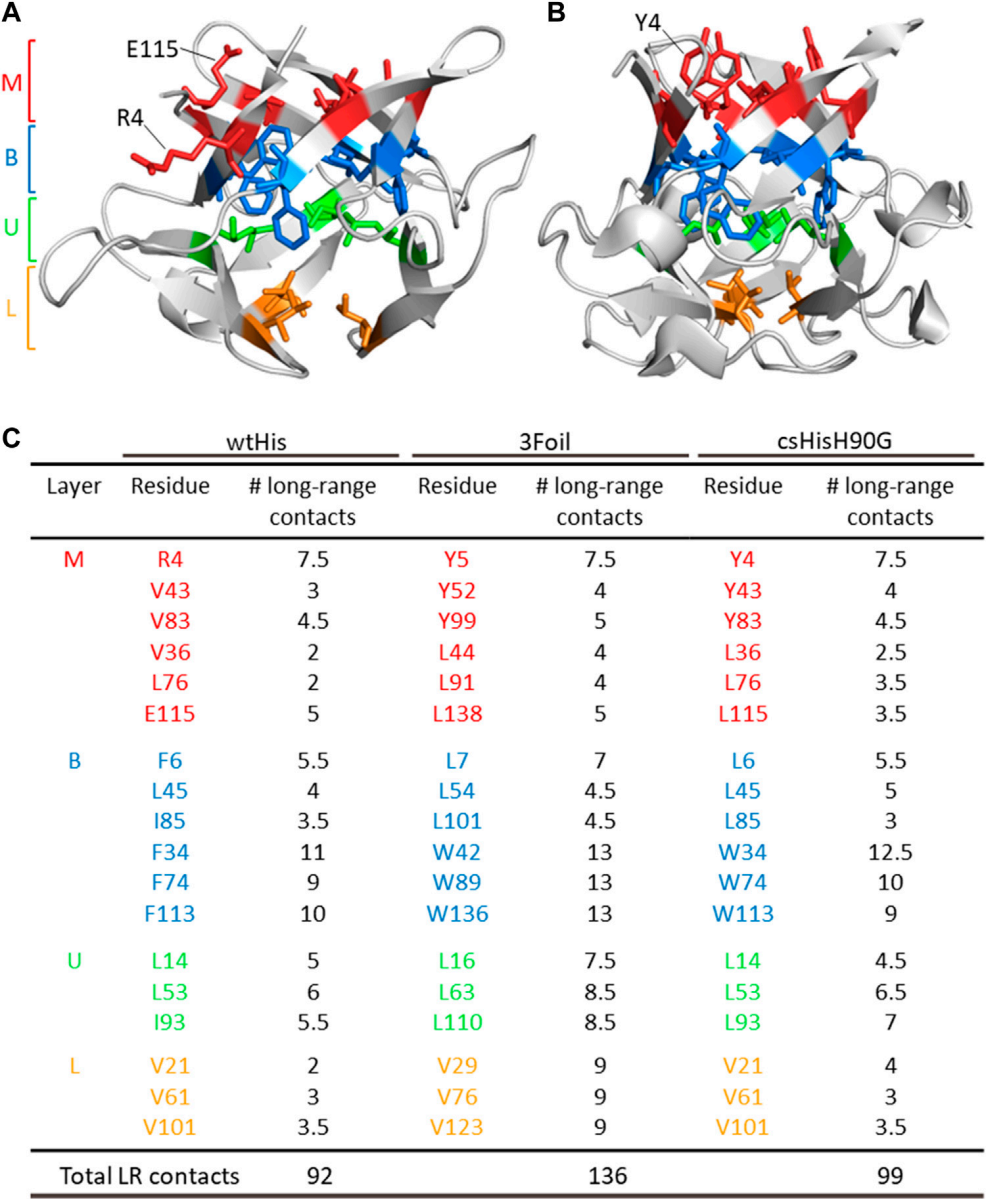
ThreeFoil core residues make significantly more long-range contacts than hisactophilin core residues. **(A)** Hisactophilin (wtHis) and **(B)** ThreeFoil (3Foil) conserved core residues are colored by layer (Middle layer of the β-barrel, M; Bottom layer of the β-barrel, B; Upper layer of the β-hairpin, U; Lower layer of the β-hairpin, L) according to their position in the β-trefoil fold (panel C) (Murzin et al., 1992). **(C)** Conserved core residues are listed for wtHis, 3Foil, and core-swapped hisactophilin (csHisH90G), where 11 of 18 hydrophobic residues differ between proteins. Mini-core residues were not considered during the design of csHisH90G and are not listed. Long-range contacts made between two core residues are counted as 0.5 long-range contacts per residue. The number of core residue long-range contacts is markedly higher in 3Foil relative to wtHis. Note that the specific contacts made by a given residue may differ between proteins (e.g., wtHis R4, which points toward solvent **(A)**, makes different contacts than 3Foil Y5, which points into the protein core **(B)**). Tryptophan residues used to monitor fluorescence in 3Foil and csHisH90G are located in the B layer.

**FIGURE 3:**
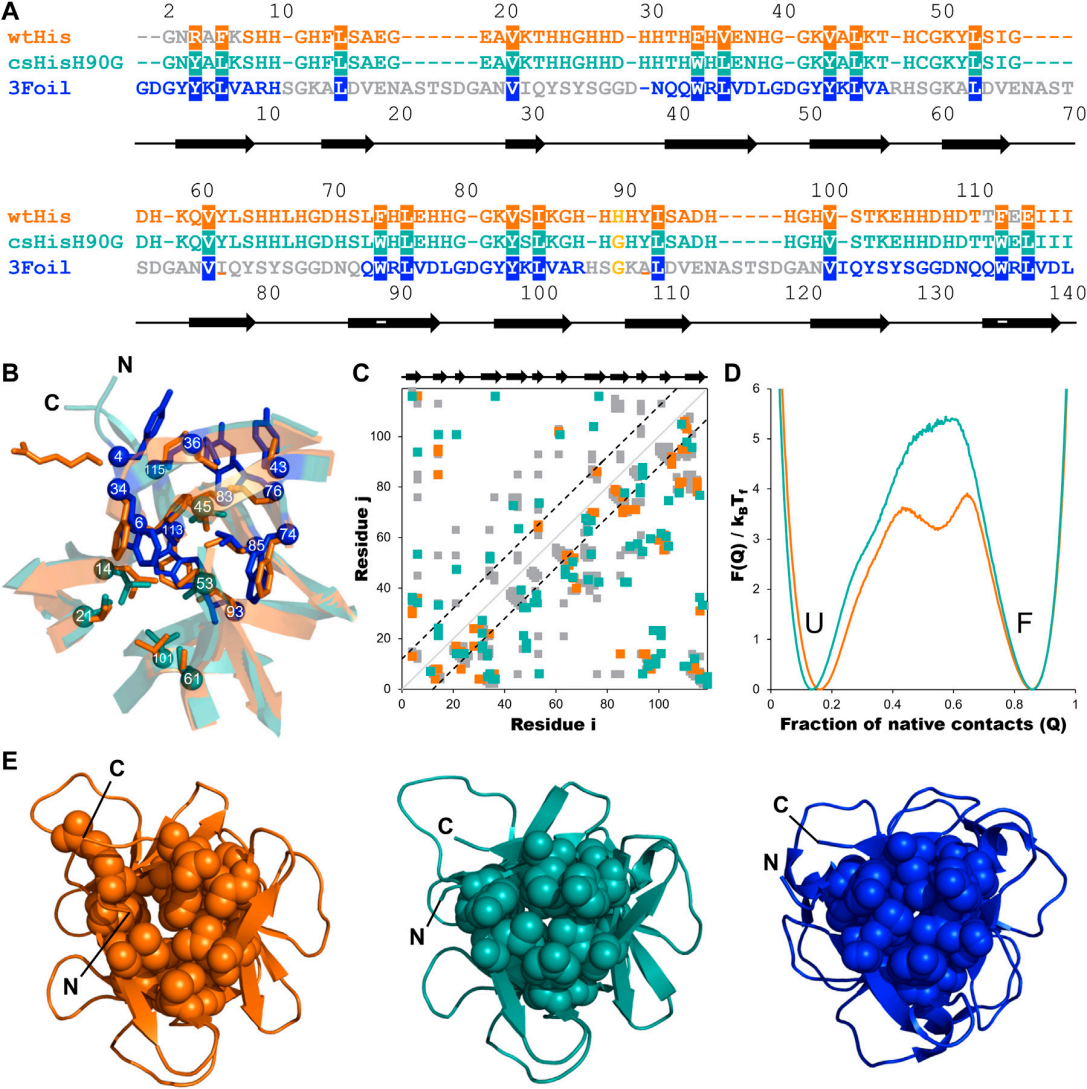
Engineering long-range intramolecular contacts between core residues enhances the hisactophilin unfolding free energy barrier. Hisactophilin (wtHis; orange) core residues were replaced with those of ThreeFoil (3Foil; blue) to give the core-swapped hisactophilin variant csHisH90G (cyan). **(A)** Sequences for wtHis, csHisH90G, and 3Foil are given as a structure-based sequence alignment with the 18 conserved core residues targeted for engineering highlighted. The thermodynamically stabilizing point mutation H90G is yellow. Secondary structure for wtHis and csHisH90G is indicated below the alignment, with β-strands represented as arrows. Residue numbers for wtHis and csHisH90G are given above the alignment. Residue numbers for 3Foil are given below the alignment. wtHis and 3Foil residues that were excluded from structural templates used in Rosetta Comparative Modeling to generate the csHisH90G model are in grey. Residues used to monitor changes in protein fluorescence (Y62 and Y92 in wtHis; W34, W74, and W113 in csHisH90G; W42, W89, and W136 in 3Foil) are underlined. **(B)** wtHis and csHisH90G are overlaid to illustrate mutated core residues. wtHis core residues are given in orange. csHisH90G core residues derived from 3Foil are shown in blue, and csHisH90G core residues that are unchanged from wtHis (i.e., equivalent 3Foil residues already had the same amino acid identity as wtHis) are given in cyan. C_α_ atoms are shown as spheres and are numbered according to the alignment to wtHis given in **(A)**. Loop residues are removed for simplicity. **(C)** Difference contact map for wtHis and csHisH90G. Contacts common to both proteins are shown in grey. The top left portion shows contact pairs made by core residues to any other residue. The bottom right portion shows all residue pairs for each protein. Long-range contacts, in which residues *i* and *j* are more than 11 residues apart in the primary sequence, are all contacts outside of the back dashed lines. Secondary structure is indicated above the contact map. C_α_ contact maps were generated using the Shadow map algorithm available through SMOG2 using default parameters (i.e., 6 Å maximum contact cutoff and 1 Å atom occlusion) (Noel et al., 2012; 2016). All simulations for wtHis and csHisH90G were completed using SMOG2 shadow maps. **(D)** wtHis and csHisH90G unfolding free energy barriers were simulated using C_α_-SBMs. Simulations were run at each protein’s folding temperature, and unfolding free energy barriers were solved using the Boltzmann reweighting method described by Gosavi *et al.* (2006). Unfolding free energy barriers are given along the reaction coordinate Q, the fraction of native contacts. The unfolded (U) and folded (F) states are indicated. The unfolding free energy barrier predicted for csHisH90G is 1.5 k_B_T_f_ larger than that predicted for wtHis. Unfolding free energy barrier heights are given in Table 1. **(E)** Native structures for wtHis (orange, left), csHisH90G (cyan, middle), and 3Foil (blue, right) are given looking down the β-barrel (i.e., the N- and C-termini facing out of the page) with the 18 conserved core residues shown in space-filling representation. Improved core packing density is evident from wtHis to csHisH90G and from csHisH90G to 3Foil.

Here, we describe the design and characterization of a hisactophilin variant, core-swapped hisactophilin (csHisH90G), mutated to contain 3Foil core residues (Figures 2, 3A, 3B). The goal of this core-swapped design is two-fold: 1) to assess using LRO, ACO, and C_α_-SBM unfolding free energy barriers as predictive measures for rationally designing kinetic stability; and 2) to test enhancing protein kinetic stability within the confines of the protein’s existing chain length and fold (Thirumalai, 1995). Using LRO, ACO, and C_α_-SBM simulations, we predict a moderate increase in csHisH90G topological complexity and unfolding free energy barrier height, both indicating improved kinetic stability relative to wtHis. Based on these predictions, we experimentally expressed and purified a single csHisH90G design, which we show to be well-behaved and well-folded *in vitro.* Experimental kinetic folding and unfolding measurements confirm that csHisH90G displays greater kinetic stability than its parent protein, pseudo wild type hisactophilin H90G (HisH90G). Further, kinetic data shows that csHisH90G displays folding behavior intermediate to wtHis and 3Foil. We discuss the advantages and limitations of using LRO, ACO, and C_α_-SBM simulations for protein kinetic stability design compared to prevailing methods. Finally, we propose that engineering protein cores, either by swapping conserved hydrophobic core residues in homologous protein folds or using *de novo* core packing software, may offer a feasible and robust strategy for improving kinetic stability in designed proteins.

## 2 Materials and methods

### 2.1 *In silico*

LRO, ACO, and C_α_-SBM unfolding free energy barriers were used as predictive values to guide kinetic stability design in wtHis and 3Foil variants. *In silico* methods focused primarily on identifying structurally equivalent residues in wtHis and 3Foil that, when swapped into wtHis, sufficiently increase the LRO, ACO, and unfolding free energy barrier heights in C_α_-SBM simulations and, thus, predict increased kinetic stability of hisactophilin variants (Figure 3; Table 1).

**TABLE 1:** Predicted and experimental protein unfolding kinetics.

wtHis		csHisH90G	3Foil
LRO	4.1	4.5	6.2
$k_{u,\;{\;C}_{mid}\;\left(ACO\right)}$ (s^−1^)^a^	4.3 × 10^−1^	1.0 × 10^−1^	9.8 × 10^−5^
ACO	12.2	13.1	22.6
$k_{u,\;{\;C}_{mid}\;\left(ACO\right)}$ (s^−1^)^b^	3.6 × 10^−1^	1.3 × 10^−1^	1.5 × 10^−6^
Free energy barrier height ( $\Delta G_{u}/k_{B}T$ )^c^	3.9	5.4	17.0^d^
Experimental $k_{u,\;{\;C}_{mid}\;}$ (x10^−3^ s^−1^)	**6.7** ± **3.1** ^e^	3.4 ± 1.8	1.9 ± 0.8 × 10^−5^
	3. 4± 1.1		

^a^ $k_{u,\;{\;C}_{mid}\;\left(LRO\right)}$ is calculated using the linear relation $k_{u,\;{\;C}_{mid}}=-1.7\left(LRO\right)+6.6$ for two-state β-proteins at the transition midpoint (Broom et al., 2015a).

^b^ $k_{u,\;{\;C}_{mid}\;\left(ACO\right)}$ is calculated using the linear relation $k_{f,\;{\;C}_{mid}}=-0.52\left(ACO\right)+5.9$ for two-state β-proteins at the transition midpoint (Broom et al., 2015a).

^c^ Free energy barrier heights were generated from C_α_-SBM, folding simulations at the protein folding temperature, the simulation temperature equivalent to the transition midpoint (see *Methods*).

^d^ C_α_-SBM, folding simulations for 3Foil are given in the Supplementary Material.

^e^ Experimental $k_{u,\;{\;C}_{mid}\;}$ for HisH90G, the pseudo wild type for csHisH90G.

#### 2.1.1 Identifying target residues for kinetic stability design

Residues *i* and *j* in a given protein are said to be in contact if a pair of heavy atoms belonging to residues *i* and *j* are in close proximity in the protein’s folded state. In the C_α_-SBM, used herein, a contact between a pair of atoms is projected onto the C_α_ atoms of the corresponding residues *i* and *j* (Clementi et al., 2000). All contacting residue pairs are compiled in a list (see Supplementary Material). A contact map is a symmetric plot of this list with both *x* and *y*-axes denoting residue numbers. Colored boxes are marked on this plot at (*i*, *j*) and (*j*, *i*) when a contact is present between residues *i* and *j* (Figure 3C). Contact maps were generated for energy-minimized structures of wtHis (PDB ID: 1HCD) and 3Foil (PDB ID: 3PG0) using the C_α_ Shadow algorithm available on the SMOG2 web server (Noel et al., 2012; Noel et al., 2016). Shadow maps used default parameters of a 6 Å maximum contact cutoff and 1 Å atom “shadowing” radius (Noel et al., 2012; Noel et al., 2016). wtHis and 3Foil were aligned using a sequence-based structure alignment, and equivalent residues were identified (Figure 3A). Residue pairs in wtHis and 3Foil contact maps were compared to identify conserved networks of interacting, structurally equivalent residues in which wtHis residues make fewer contacts than those of 3Foil (Figure 3; see *Results* for details). Identification of long-range interaction networks was prioritized over local interactions since alteration of long-range interactions is captured in ACO and LRO measures, while local interactions are only represented in ACO. This is because ACO is the average sequence separation between contacting heavy atoms calculated as:

$$ACO=\frac{1}{N_{c}}\overset{N_{c}}{\underset{i,j}{\sum }}\left|i-j\right|$$ (1)

where $N_{c}$ is the total number of contacts between heavy atoms, $\left|i-j\right|$ is the sequence separation in residues for a given contacting pair of atoms in residues *i* and *j* with $\left|i-j\right|>1$ , and contacts are only considered between heavy atoms less than 6 Å apart (Ivankov et al., 2003), while LRO is calculated as:

$$LRO=\frac{1}{L}\overset{R_{c}}{\underset{i,j}{\sum }}n_{i,j}$$ (2)

where *L* is the protein chain length in amino acid residues, $R_{c}$ is the total number of contacting residue pairs *i* and *j*, $n_{i,\;j}$ is equal to 1 when $\left|i-j\right|\geq 12$ and 0 otherwise, and a residue pair is considered to be in contact when heavy atoms in residues *i* and *j* are within 6 Å (Gromiha and Selvaraj, 2001; Broom et al., 2015a). Additionally, LRO provides a stronger, more linear correlation for proteins of variable size since it is normalized to chain length (Broom et al., 2015b). 3Foil and wtHis core residues were identified as promising targets for modulating LRO and ACO in a residue-swapped wtHis/3Foil hybrid since wtHis core residues make significantly fewer long-range contacts than 3Foil core residues (Figure 2).

The hisactophilin point mutant H90G (HisH90G) was identified in previous equilibrium denaturation experiments to be thermodynamically stabilized compared to wtHis (MacKenzie et al., 2022). Here, HisH90G was used as a pseudo wild type parent protein for the core-swap design, and the H90G point mutation was included in the core-swapped hisactophilin variant (see *Results*).

#### 2.1.2 Generating the core-swapped model

Ten structural models of csHisH90G were generated using Robetta Comparative Modeling (Chivian et al., 2003; Song et al., 2013) with wtHis and 3Foil structures as templates. The sequence used to generate the csHisH90G models, wherein HisH90G core residues are mutated to structurally equivalent 3Foil core residues, is given in Figure 3A. Template alignments were modified such that csHisH90G core residues and β-strands 1 and 12 (residues 1–7 and 112–115, respectively) were modeled after 3Foil residues only, and csHisH90G hairpin cap residues (residues 11–29, 48–73, and 89–100) were modeled after hisactophilin residues only (Figure 3A). Models with buried Y4/43/83 hydroxyl groups were discarded due to the high energy cost of burying the polar group in the protein’s hydrophobic core. LRO and ACO scores were calculated for each model, and outliers were identified using the interquartile range method and discarded. Remaining csHisH90G models all displayed increased LRO and ACO scores compared to wtHis, as expected. Finally, models were assessed using PROCHECK (Laskowski et al., 1993; Laskowski et al., 1996) and MolProbity (Williams et al., 2018) Ramachandran scores, and the model with the most favorable Ramachandran score was chosen as the structural model for csHisH90G for all subsequent computational methods.

#### 2.1.3 Predicting the unfolding rate constant of csHisH90G

csHisH90G’s unfolding rate constant was predicted using the linear correlations for the unfolding rate constant and LRO or ACO reported by Broom et al. (2015a) for β proteins (Table 1). The linear correlation for LRO and the unfolding rate constant at the transition midpoint is given by:

$$k_{u,\;C_{mid}}=-1.70\left(LRO\right)+6.6$$ (3)

where $k_{u,\;C_{mid}}$ is the unfolding rate constant at the transition midpoint. The linear correlation for ACO and the unfolding rate constant at the transition midpoint is given by:

$$k_{u,\;C_{mid}}=-0.52\left(ACO\right)+5.9$$ (4)

Experimental structural information is unavailable for HisH90G. However, LRO, ACO, and C_α_-SBMs are based on contact maps, and the H90G point mutation has little effect on the hisactophilin contact map. Specifically, H90 makes only two long-range intramolecular contacts (H90, K104 and H90, E105) and one short-range contact (K86, H90) in wtHis, and G90 makes identical contacts in the structural model for csHisH90G. Thus, the structural substitution of wtHis for HisH90G is reasonable for LRO, ACO, and C_α_-SBM simulation predictions. It should, however, be noted that sequence specific effects (e.g., secondary-structural propensities) due to the H90G mutation that may affect hisactophilin folding are not captured in LRO, ACO, or C_α_-SBM methods.

#### 2.1.4 Predicting the free energy barrier of unfolding using protein folding simulations

All simulations were carried out using the GROMACS v.4.5.4 software package (Bekker et al., 1993; Berendsen et al., 1995; Lindahl et al., 2001; Van Der Spoel et al., 2005; Hess et al., 2008). GROMACS geometry and topology files were generated for wtHis and csHisH90G using the AMBER99SB-ILDN force field and TIP3P water model (Jorgensen et al., 1983; MacKerell et al., 1998; Lindorff-Larsen et al., 2010). All protein hydrogens were ignored. Solvent molecules were replaced with Na^+^ or Cl^−^ ions until the system reached net neutral charge. Energy minimization simulations were performed for 2,000 steps using the method of steepest descent. Energy-minimized wtHis and csHisH90G structures were used in subsequent C_α_-SBM simulations.

Protein folding for wtHis and csHisH90G was investigated using C_α_-SBM simulations. Proteins fold on a biologically reasonable timescale because of a funnel-shaped energy landscape in which interactions (or contacts) present in the native state of the protein are more stabilizing than any non-native interactions that occur during protein folding (Bryngelson et al., 1995; Wolynes et al., 1995; Onuchic et al., 1997; Onuchic and Wolynes, 2004; Dill and Maccallum, 2012). Structure-based models (SBMs) encode this funnel in their potential energy functions by ignoring attractive non-native interactions and encoding attractive native interactions through inter-residue contacts calculated from the native structure. The coarse-grained C_α_-SBM used here to simulate wtHis, csHisH90G, and 3Foil has previously been used successfully to simulate the folding of several proteins (Clementi et al., 2000; Chavez et al., 2004; Gosavi et al., 2006; Gosavi et al., 2008; Hills and Brooks, 2009; Hyeon and Thirumalai, 2011; Gosavi, 2013; Broom et al., 2015b; Giri Rao and Gosavi, 2018). The exact form of the potential energy function of this C_α_-SBM is given elsewhere (Clementi et al., 2000). Geometry, topology, table, and parameter files required for C_α_ coarse-grained simulations were obtained from the SMOG2 webserver (Noel et al., 2012; Noel et al., 2016). Contact maps were generated using the same criteria given above.

C_α_-SBM simulations were performed using a stochastic dynamics integrator with a 0.0005 ps time step. All simulations were performed using the NVT ensemble. Proteins were simulated at their respective folding temperatures (T_f_), which is defined as the temperature at which the folded and unfolded states are equally populated and folding transitions occur from both the unfolded and folded states to ensure reasonable sampling of the transition state ensemble (TSE). Unfolded protein geometry files for wtHis and csHisH90G were obtained by running short, high temperature (*T* = 230 K) simulations. Note that since these are coarse-grained simulations, simulation temperatures do not directly correspond to experimental temperatures. Preliminary simulations were initiated using the native protein geometry and the unfolded protein geometry for each temperature and performed for 1 × 10^8^ time steps. Folding temperatures for wtHis and csHisH90G were determined by performing preliminary simulations over iteratively smaller temperature ranges until folding transitions (i.e., the folded state transitioned to the unfolded state or *vice versa*) occurred from folded and unfolded structures and the populations of both states were approximately equal. Production runs were performed at the T_f_ for a total of 2 × 10^10^ time steps. Folding simulations for 3Foil, which required enhanced sampling due to its unusually large free energy barrier, are described in the Supplementary Material.

Since through-space attractive interactions are primarily encoded in the C_α_-SBM through native contacts, the fraction of native contacts (Q) is often used as a progress coordinate (Clementi et al., 2000; Chavez et al., 2004). Here, we plot the unfolding free energy barrier as a function of Q (Figure 3D). The number of formed contacts is calculated for every simulation snapshot. A contact is said to be formed if the distance between the contacting residues is less than 1.2 times their distance in the folded structure. The Q of a given snapshot is the number of contacts formed in that snapshot divided by the total number of native contacts. Snapshots are then pooled and binned based on their Q into a histogram P(Q). The free energy F(Q) is then equal to -ln[P(Q)] and is plotted as a function of Q. This plot has at least two minima: one at low Q that represents the unfolded minimum, and one at high Q that represents the folded minimum. The free energy barrier separating these minima is the unfolding free energy barrier. To compare free energy barriers of different proteins, simulations of each protein are reweighted such that the folded and unfolded have the same free energy, which is set to 0 (Figure 3D). We assume free energy barriers to be experimentally distinguishable if their heights differ by ∼2 k_B_T_f_ (Onuchic and Wolynes, 2004; Gosavi, 2013).

In order to understand any changes in the folding pathway, we also plotted average contact maps of wtHis, csHisH90G, and 3Foil near the transition state ensemble (Q = 0.40). To plot the wtHis and csHisH90G contact maps, all simulation snapshots at the required Q were pooled. In each snapshot, the value of a formed contact is set to 1 and the value of an unformed contact is set to 0. The value of a contact in an average contact map calculated at Q is the value of that contact averaged over all snapshots at the Q. In the average contact map, the boxes marking the contacts are colored according to their value. Consequently, the average contact map is a visual representation of the average partially folded structure of the protein at Q. Average contact maps for 3Foil are described in the Supplementary Material.

### 2.2 Experimental

#### 2.2.1 Protein expression

wtHis and pseudo wild type HisH90G were expressed using the pHW plasmid in *Escherichia coli* BL21 cells as previously described (Wong et al., 2004). csHisH90G was expressed using the pET28a^+^ expression vector in *E. coli* BL21 (DE3) cells with pLysS. All cell strains were inoculated into 2TY media and grown at 37°C with shaking for approximately 3 h. Upon reaching OD_600_ 0.7, cells were induced with 0.5 mM IPTG. Post induction, cells were grown at 25°C with shaking for 20–24 h. Cells were harvested at 5000 g, and cell pellets were stored at −80°C until cell lysis.

#### 2.2.2 Cell lysis and protein purification

Cells were resuspended in 50 mM Tris buffer pH 8.0 with 0.1 M NaCl, 1 mM MgCl_2_, and 0.1 mM PMSF. Once homogenous, DNase I was added to the resuspension, and cells were lysed at >17,000 psi for 5 min using the Emulsiflex®-C5 High Pressure Homogenizer (AVESTIN, Inc, ON, Canada). Lysate was centrifuged twice at 20,000 rpm (47,850 g) for 22 min at 4°C, and the supernatant was filtered using a 0.45 μm syringe filter.

wtHis, HisH90G, and csHisH90G bind Ni-NTA resin without the use of a His tag due to their high histidine content (31 of 117 residues in wtHis). As such, wtHis, HisH90G, and csHisH90G were purified *via* nickel immobilized metal affinity chromatography using Profinity IMAC resin (Profinity IMAC, BioRad Laboratories Inc, CA, United States) using a BioRad low-pressure chromatography system (BioRAD BioLogic LP, BioRad Laboratories Inc, CA, United States). The nickel affinity column was equilibrated with 50 mM Tris buffer pH 8.0 with 0.1 M NaCl, 1 mM MgCl_2_, and 0.1 mM PMSF. Following loading of filtered lysate, the column was washed with 50 mM sodium phosphate buffer pH 6.3 with 0.1 M NaCl, 20 mM imidazole, and 0.1 mM PMSF (MacKenzie et al., 2022) (Profinity™ IMAC, BioRad Laboratories Inc, CA, United States). Then, wtHis, HisH90G, or csHisH90G was eluted using 50 mM sodium phosphate buffer pH 6.3 with 0.1 M NaCl, 0.25–0.5 M imidazole, and 0.1 mM PMSF. Purified protein was dialyzed against 25 mM ammonium carbonate pH 8.9 using 10 kDa molecular cutoff dialysis tubing (Repligen Spectra/Por 6 tubing, Spectrum Laboratories Inc, CA, United States). Protein was concentrated to 5–10 mg/mL using an Amicon^®^ Stirred Cell (EMD Millipore Corporation, MA, United States) and a 10 kDa molecular weight cut-off membrane (Ultra Cel^®^ 10 kDa Ultrafiltration Discs, EMD Millipore Corporation, MA, United States). Following concentration, protein was lyophilized and stored at −80°C.

#### 2.2.3 Guanidine hydrochloride equilibrium denaturation

**FIGURE 4:**
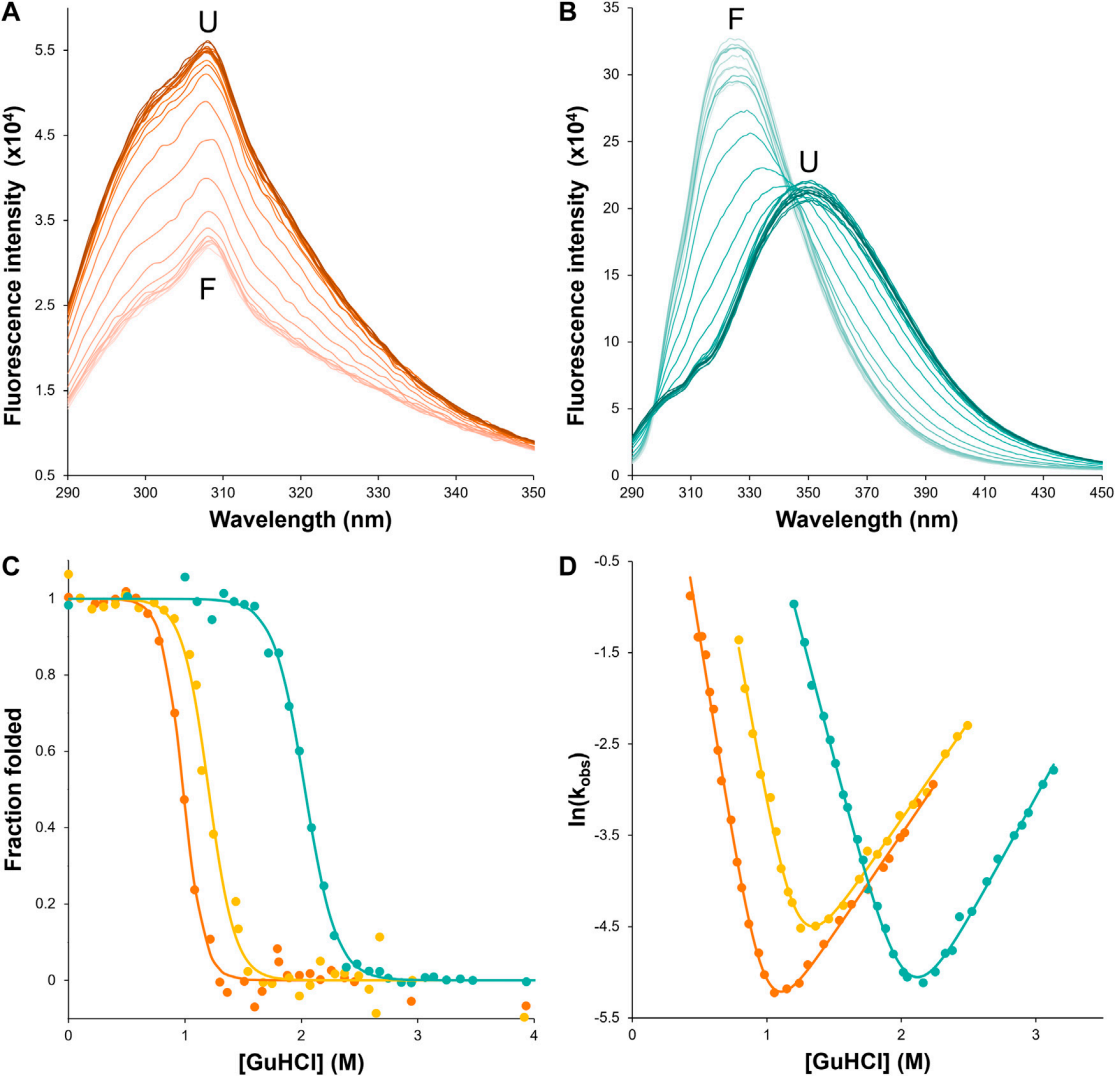
csHisH90G displays successful kinetic stabilization over HisH90G. Fluorescence emission spectra of unfolding equilibria for **(A)** wtHis and **(B)** csHisH90G in 0–4 M GuHCl (darker color indicates higher denaturant concentration). csHisH90G shows a pronounced red shift from the folded state (F) at 325 nm to the unfolded state (U) at ∼350 nm. **(C)** Fluorescence-monitored GuHCl denaturation curves displayed as the fraction of folded protein for wtHis (orange), HisH90G (yellow), and csHisH90G (cyan). Solutions contained 50 mM potassium phosphate pH 7.7, 0–4 M GuHCl, 1 mM DTT, and 10 μM protein for wtHis and HisH90G or 6 μM protein for csHisH90G. csHisH90G is significantly thermodynamically stabilized compared to wtHis and HisH90G. All samples were equilibrated at 27°C for at least 10 half-lives. **(D)** Chevron plots for observed folding and unfolding rate constants for wtHis, HisH90G, and csHisH90G at 27°C. wtHis and HisH90G kinetics were monitored at 306 nm with excitation at 277 nm. csHisH90G kinetics were monitored at 314 nm with excitation at 280 nm. csHisH90G is kinetically stabilized compared to its parent protein HisH90G. Data points in **(C)** and **(D)** are for single measurements. The results are representative of at least two independent experiments. Values for equilibrium and kinetic fits are given in Table 2. Models used to fit equilibrium and kinetic data are given in Methods.

Lyophilized wtHis, HisH90G, or csHisH90G was dissolved in 50 mM potassium phosphate buffer pH 7.7 with 1 mM DTT to a final concentration of ∼150 μM wtHis and HisH90G protein stocks were diluted to 10 μM and csHisH90G was diluted to 6 μM in various concentrations of guanidine hydrocholoride (GuHCl) ranging from 0 to 7.5 M in 50 mM potassium phosphate buffer. All samples were equilibrated at 27°C for at least ten half-lives of unfolding. Equilibrium fluorescence scans were collected for each sample using a PTI QuantaMaster™ Series fluorometer (QM-0875-21 Modular Research Fluorometer, Horiba Scientific, ON, Canada). wtHis and HisH90G unfolding equilibria were measured using tyrosine fluorescence at 306 nm with excitation at 277 nm (Figures 4A, C) (Wong et al., 2004). csHisH90G unfolding equilibria were measured using tryptophan fluorescence at 314 nm with excitation at 280 nm (Figures 4B, C) (Broom et al., 2012). All scans were done with 1 nm excitation and 5 nm emission slit widths. The resulting curves were fit to a linear extrapolation model:

$$Y=\left(Y_{N}+S_{N}\left[GuHCl\right]\right)-\frac{\left(\left(Y_{N}+S_{N}\left[GuHCl\right]\right)-\left(Y_{U}+S_{U}\left[GuHCl\right]\right)\right)\left(e^{\frac{{∆G}_{U-F}-m_{eq}\left[GuHCl\right]}{RT}}\right)}{1+e^{\frac{{∆G}_{U-F}-m_{eq}\left[GuHCl\right]}{RT}}}$$ (5)

where *Y* is the optical signal of the native (*N*) or unfolded (*U*) state, *S* in the [GuHCl]-dependence of the optical signal, *ΔG*_*U-F*_ is the free energy of unfolding in water, *m*_*eq*_ is the [GuHCl]-dependence of *ΔG*_*U-F*_, *R* is the gas constant 1.987 cal K^−1^ mol^−1^, and *T* is the temperature in Kelvin (Figure 4C). The data are well fit by the 2-state unfolding model. The fitted experimental *m* values decrease with increasing $C_{mid}$ for the variants studied here, which is consistent with the known non-linear denaturant-dependence of stability (Liu et al., 2001; Wong et al., 2004). Values for equilibrium fits are given in Table 2.

**TABLE 2:** Equilibrium and kinetic parameters for wtHis, HisH90G, csHisH90G, and 3Foil.

		wtHis	HisH90G	csHisH90G	3Foil^a^
$C_{mid}$ (M)	*Eq*	0.98 ± 0.01	1.20 ± 0.02	2.03 ± 0.01	
	*Kin*	0.98 ± 0.05	1.21 ± 0.09	2.03 ± 0.08	0.79 ± 0.04
$m_{eq}$ (kcal mol^−1^ M^−1^)	*Eq*	6.66 ± 1.09	5.25 ± 1.10	4.39 ± 0.47	6.66 ± 1.09
	*Kin*	6.74 ± 0.17	6.29 ± 0.23	4.99 ± 0.10	9.42 ± 0.23
${\Delta G}_{U-F}$ (kcal mol^−1^)	*Eq*	6.55 ± 1.10	6.32 ± 1.33	8.95 ± 0.97	
	*Kin*	6.61 ± 0.16	7.63 ± 0.27	10.13 ± 0.19	7.41 ± 0.16
$k_{u}^{H_{2}O}$ (x10^−4^ s^−1^)		4.0 ± 0.5	5.1 ± 0.8	0.2 ± 0.03	2.8 ± 0.1 × 10^−6^
$m_{u}$ (kcal mol^-1^ M^-1^)		1.29 ± 0.05	1.27 ± 0.05	1.60 ± 0.04	3.20 ± 0.03
$k_{f}^{H_{2}O}$ (s^−1^)		26.0 ± 3.5	182. 3± 54.1	346.4 ± 50.5	7.0 ± 0.4 × 10^−5^
$m_{f}$ (kcal mol^-1^ M^-1^)		−5.46 ± 0.12	−5.02 ± 0.19	−3.39 ± 0.06	−6.22 ± 0.20
$k_{u,\;C_{mid}}$ (x10^−3^ s^−1^)		3.4 ± 1.1	6.7 ± 3.1	3.4 ± 1.8	1.9 ± 0.8x10^−5^
$\beta_{T}$		0.81 ± 0.04	0.80 ± 0.06	0.68 ± 0.02	0.66 ± 0.04

Error estimates were obtained from the fitting program Origin 2018. See Figure 4 and *Methods*.

^a^ 3Foil kinetics were obtained using GuSCN activity (Broom et al., 2015b). Equilibrium data could not be measured for 3Foil due to its extreme kinetic stability.

#### 2.2.4 GuHCl refolding and unfolding kinetics

Kinetic unfolding and refolding experiments were carried out using manual mixing on a PTI QuantaMaster™ Series fluorometer. For refolding experiments, lyophilized protein was dissolved in concentrated buffered GuHCl (∼8 M) to ∼150 μM. For unfolding experiments lyophilized protein was dissolved in phosphate buffer to ∼150 μM. Protein stocks were diluted to 10 μM (wtHis or HisH90G) or 6 μM (csHisH90G) at various GuHCl concentrations ranging approximately 1 M GuHCl on either side of the kinetic midpoint. Mixing dead times were ∼2–5 s. Sample fluorescence was measured for at least 10 half-lives, using the same fluorometer settings as for equilibrium experiments (above). The kinetic rate constants were obtained by fitting the data to either a single exponential model:

$$Y=A\left(e^{-t/t_{1}}\right)+Y_{0}$$ (6)

or a single exponential model with a linear drift:

$$Y=A\left(e^{-t/t_{1}}\right)+Y_{0}+dt$$ (7)

where *A* is the amplitude of the change in fluorescence, *t* is time in seconds, *t*_*1*_ is the inverse of the rate constant, *k*, *Y*_*0*_ is the intensity of the fluorescence at *t* = 0 s, and *d* is the drift. Rate constants were then fit to a 2-state model as previously described (Liu et al., 2001) given by:

$$\ln\left(k_{obs}\right)=\ln\left({k_{f}^{H_{2}O}e}^{\left(\frac{m_{f}\left[GuHCl\right]}{RT}\right)}+{k_{u}^{H_{2}O}e}^{\left(\frac{m_{u}\left[GuHCl\right]}{RT}\right)}\right)$$ (8)

where *k*_*obs*_ is the measured rate constant, $m_{f}/RT$ and $m_{u}/RT$ are the linear [GuHCl]-dependences of the folding and unfolding rate constants, respectively (Figure 4D), and $k_{f}^{H_{2}O}$ and $k_{u}^{H_{2}O}$ are the folding and unfolding rate constants in water, respectively. The equilibrium *m*-value ( $m_{eq}$ ) was calculated by:

$$m_{eq}=m_{u}-m_{f}$$ (9)

and reflects the total increase in solvent accessible surface area between the protein’s folded and unfolded states. The β-Tanford value (β_T_) for folding reflects the change in solvent accessible surface area of the transition state relative to the unfolded state, and is given by:

$$\beta_{T}=m_{f}/m_{eq}$$ (10)

where a value of 1 indicates a native-like transition state and a value of 0 indicates an unfolded-like transition state. The equilibrium Gibbs free energy of unfolding was calculated by:

$${\Delta G}_{U-F}=-RT\;\ln\left(\frac{k_{u}^{H_{2}O}}{k_{f}^{H_{2}O}}\right)$$ (11)

Measured and calculated kinetic parameters are given in Table 2.

## 3 Results

### 3.1 Ensuring sufficient thermodynamic stability in the parent protein

While improving wtHis kinetic stability is the primary objective of our design, thermodynamic stability must also be considered. Previous equilibrium denaturation and kinetic studies on hisactophilin show that the point mutation H90G is thermodynamically stabilizing (MacKenzie et al., 2022). Glycine is highly conserved in this position in all three trefoils of this symmetric fold in other β-trefoil proteins (Ponting and Russell, 2000). Glycine is also present at the equivalent positions in wtHis’ other two trefoils, and 3Foil has glycine in all three structurally equivalent positions (Figure 3A) (Ponting and Russell, 2000; Broom et al., 2012). Thus, to improve the thermodynamic stability of our parent protein and to increase the probability of expressing a well-folded core-swapped protein, we use the H90G point mutant as a stabilized pseudo wild type (HisH90G) from which to engineer our core-swapped design.

### 3.2 3Foil core residues promote long-range contact formation and increase topological complexity in hisactophilin

To increase kinetic stability in wtHis, we sought to engineer additional long-range intramolecular contacts to increase the topological complexity of wtHis, as measured by LRO, ACO, and free energy barrier heights from C_α_-SBM simulations. Toward this end, we compared intramolecular contacts in wtHis to those of 3Foil, which displays extreme kinetic stability and shares a common fold with wtHis (Broom et al., 2015b) (Figures 3A, E). Here, we focus on differences in contacts made by 18 residues that are conserved as core residues in β-trefoils (Murzin et al., 1992; Ponting and Russell, 2000). 3Foil core residues contribute 136 long-range contacts to its LRO, while those of wtHis contribute only 92 (Figure 2). 3Foil and wtHis differ at 11 of the 18 core residues (Figures 3A, B) and display markedly different core packing (Figures 3B,E). Notably, R4 and E115 in wtHis twist away from the hydrophobic core to point toward solvent (Hanakam et al., 1996) (Figures 2A, B; Figures 3B, E). In comparison to 3Foil, the wtHis core is largely composed of relatively small residues like valine, which are less densely packed that in other β-trefoil proteins (Lee and Blaber, 2011; Broom et al., 2012; Terada et al., 2017; Blaber, 2021). Together, wtHis’ unusual R4 and E115 backbone conformations and diminished core packing create a cavity through the protein core with a cavity volume of 65 Å^3^, as calculated using CASTp (Tian et al., 2018) (Supplementary Figure S1). In contrast, 3Foil’s core is closely packed with no detectable cavity and contains larger tyrosine and tryptophan residues that all point inward to make long-range interactions throughout the core (Figure 3E). Further, 3Foil core residues have a combined volume of 3.2 × 10^3^ Å^3^, whereas equivalent residues in wtHis have ∼10% decreased volume of 2.9 × 10^3^ Å^3^ (Perkins, 1986). Reducing the core cavity volume and bringing core residue side chains into closer proximity is expected to achieve the formation of additional long-range contacts across the protein core and increase protein topological complexity. As such, replacing wtHis core residues with those of 3Foil is expected to increase LRO, ACO, and kinetic stability in hisactophilin.

Incorporating 3Foil core residues into wtHis markedly increases core packing density, as illustrated by the decrease in core cavity size in space-filled models from wtHis to csHisH90G (Figure 3E). In contrast to R4 and E115 in wtHis, the corresponding residues Y4 and L115 in csHisH90G point into the protein core, eliminating the twisted backbone conformations of wtHis and reducing the size of the cavity. Indeed, CASTp predicts only 2.57 Å^3^ of space in the csHisH90G core (Tian et al., 2018) (Supplementary Figure S1), and ProteinVolume calculates an increase in protein volume from 15.6 × 10^3^ Å^3^ in wtHis to 16.3 × 10^3^ Å^3^ in csHisH90G (Chen and Makhatadze, 2015). Introducing 3Foil core residues into wtHis concomitantly increases the number of long-range contacts made in csHisH90G relative to wtHis (Figure 3C; Supplementary Material). While several new contacts are formed between core residues, many of the new long-range interactions are made between core residue backbone groups and loop or mini-core residues (Figure 3C) (Dubey et al., 2005). Of the new contacts made between core residue side chains in csHisH90G, most do not qualify as long-range contacts according to the definition used to calculate LRO (see *Methods)*, as they are less than 12 residues apart in the primary sequence. This is owing to hisactophilin’s relatively short β2-β3 loops and tight hairpin turns, which are longer in 3Foil and other β-trefoil proteins (See Supplementary Discussion). Despite hisactophilin’s more limited capacity to form long-range contacts between core residues, contact maps for wtHis and csHisH90G show that csHisH90G gains eight long range core-core contacts and 17 additional long-range contacts between core residues and loop or mini-core residues that are not present in wtHis (see Supplementary Material). Thus, our model for csHisH90G suggests that 3Foil core residues successfully increase long-range contacts in the hisactophilin core.

Parallel to the observed enrichment of long-range contacts in csHisH90G, LRO increases from 4.1 in wtHis to 4.5 in csHisH90G (Table 1). Using the linear correlation between LRO and unfolding rate constants for two-state β proteins reported by Broom et al. (2015a), this difference in LRO predicts a 4.1-fold decrease in csHisH90G’s unfolding rate constant compared to wtHis at the transition midpoint and a corresponding 4.1-fold increase in unfolding half-life. ACO calculations also indicate that csHisH90G is kinetically stabilized compared to wtHis. csHisH90G’s ACO increases to 13.1 from 12.2 in wtHis (Table 1). The linear correlation for ACO and unfolding rates in two-state β proteins at the transition midpoint predicts that csHisH90G’s unfolding rate constant is 2.8-fold slower than wtHis (Broom et al., 2015a). Since both LRO and ACO predict higher topological complexity and slower unfolding rates for csHisH90G, we continued to investigate whether this core-swapped design increases hisactophilin kinetic stability. To obtain a higher resolution model of the change in kinetic stability, we performed C_α_-SBM simulations to model free energy barriers of unfolding for wtHis and csHisH90G.

Structure-based models (SBMs) encode a protein’s native contacts in their energy function and are useful for probing the relationship between protein folding and protein topology (Nymeyer et al., 1998; Chavez et al., 2004; Hyeon and Thirumalai, 2011). Here, we applied C_α_-SBM simulations to gain insight into wtHis and csHisH90G unfolding free energy barriers, which we use as a predictive measure for relative kinetic stability. Specifically, we used C_α_-SBM simulations to model each protein’s unfolding free energy barrier at the transition midpoint, where larger barrier heights are correlated with higher kinetic stability *in vitro* (Kramers, 1940; Chavez et al., 2004; Gosavi, 2013; Broom et al., 2015b). Using a shadow contact map with a 6 Å maximum contact distance and a 1 Å atom “shadowing” radius, wtHis is predicted to have a free energy barrier of unfolding of 3.9 k_B_T_f_ (Table 1), which is in overall agreement with previously reported barrier heights for wtHis using an alternate form of contact map that uses the same potential energy function (Gosavi, 2013; Broom et al., 2015b). C_α_-SBM folding simulations for csHisH90G predict a larger maximum unfolding free energy barrier height of 5.4 k_B_T_f_ for csHisH90G (Table 1), with an average increase in barrier height of 1.8 k_B_T_f_ over wtHis for the transition region (Q = 0.45–0.65) and a maximum barrier height difference of 2.2 k_B_T_f_ at Q = 0.54 (Figure 3D). Thus, LRO, ACO, and C_α_-SBM unfolding free energy barrier heights all predict a modest but measurable increase in protein kinetic stability for csHisH90G. We therefore proceeded to validate the design experimentally.

### 3.3 csHisH90G fluorescence suggests a 3Foil-like core

First, we assessed whether csHisH90G successfully adopts a well-folded tertiary structure with 3Foil-like core packing by comparing csHisH90G fluorescence to that of wtHis and 3Foil. csHisH90G fails to unfold in 7 M urea, so we used a stronger denaturant, guanidine hydrochloride (GuHCl). Native wtHis and HisH90G exhibit a fluorescence emission maximum at 306 nm in GuHCl (Figure 4A), in good agreement with previous equilibrium experiments performed in urea (Liu et al., 2001). Since wtHis and HisH90G lack tryptophan residues and fluorescence arises predominately from tyrosines, no shift in maximum emission wavelength is observed upon wtHis or HisH90G chemical denaturation, and protein denaturation is instead manifested by an increase in fluorescence intensity. In contrast, csHisH90G displays maximum emission at ∼325 nm in the native state and ∼350 nm in the denatured state (Figure 4B), consistent with tryptophan fluorophores going from a buried hydrophobic environment to a solvent-exposed environment upon GuHCl denaturation (Vivian and Callis, 2001). csHisH90G denaturation shows striking similarity to 3Foil, which upon unfolding also undergoes a pronounced red shift from 323 nm in the native state to ∼360 nm in the unfolded state in guanidine thiocyanate (GuSCN) (Broom et al., 2012). The unusually strong blue shift observed in native 3Foil is attributed to its densely packed core, which renders 3Foil tryptophan residues completely inaccessible to solvent (Vivian and Callis, 2001; Broom et al., 2012). The similar blue shift for csHisH90G supports the core of csHisH90G also being well-packed and resembling that of 3Foil. However, since csHisH90G is slightly less blue shifted compared to 3Foil and exhibits a less pronounced red shift upon unfolding, the csHisH90G core may be more accessible to solvent than the 3Foil core. This interpretation agrees with our structural model of csHisH90G, which shows core packing similar to, but not as close packed as, 3Foil (Figure 3E). Nevertheless, these data show that csHisH90G is well-folded *in vitro*, indicating successful engineering of 3Foil core residues into wtHis.

### 3.4 3Foil core residues enhance hisactophilin thermodynamic stability

To further validate that the csHisH90G design results in a well-behaved, cooperatively folded protein, we measured its folding equilibrium and thermodynamic stability by chemical denaturation. The fraction of folded protein with increasing GuHCl concentration is shown in Figure 4C, and fitted parameters for two-state equilibrium denaturation curves are given in Table 2. Relative stabilities are assessed by $C_{mid}$ , which is the most accurate measure of relative stability (Pace, 1986; Fersht, 1999). Folding is fully reversible for wtHis, HisH90G, and csHisH90G. The denaturation curve for HisH90G is shifted to higher GuHCl concentration compared to wtHis, with $C_{mid}$ values of 1.20 M GuHCl and .98 M GuHCl, respectively. Therefore, HisH90G has increased thermodynamic stability compared to wtHis, as expected (MacKenzie et al., 2022). Notably, csHisH90G shows significant thermodynamic stabilization compared to wtHis and HisH90G, with a $C_{mid}$ of 2.03 M GuHCl (see Supplementary Discussion). Kinetic midpoints for wtHis, HisH90G, and csHisH90G show excellent agreement with equilibrium midpoints, consistent with 2-state unfolding transitions (Table 2).

### 3.5 3Foil core residues enhance kinetic stability in csHisH90G

To assess the outcome of our kinetic stability design, we measured the folding kinetics of wtHis, HisH90G, and csHisH90G using chemical denaturation (Figure 4D). wtHis displays moderate kinetic stability in GuHCl, with an unfolding rate constant of 3.4 × 10^−3^ s^−1^ and a half-life of ∼3.5 min at the transition midpoint. The H90G point mutation decreases kinetic stability relative to wtHis, increasing the HisH90G unfolding rate constant to 6.7 × 10^−3^ s^−1^ and reducing its half-life to ∼1.7 min at the transition midpoint. We hypothesize that the molecular basis for the accelerated unfolding and folding kinetics in HisH90G may be related to favoring folding and formation of a distinctive turn-like conformation in β-trefoils, where this glycine is strongly conserved (Ponting and Russell, 2000). Molecular details of this sequence-specific effect cannot be captured by LRO, ACO, or C_α_-SBM simulations. Notably, HisH90G retains similar denaturant dependence of folding ( $m_{f}$ ) and unfolding kinetics ( $m_{u}$ ) to wtHis (Table 2), indicating that the changes in solvent accessible surface area from the folded or unfolded state to the transition state are maintained. Accordingly, the HisH90G β-Tanford ( $\beta_{T}$ ) value, which reports on the structure of the transition state, is nearly unchanged compared to wtHis (Table 2). Thus, we can conclude that despite lower kinetic stability in HisH90G, the HisH90G folding pathway and transition state are similar to those of wtHis.

csHisH90G has enhanced kinetic stability compared to HisH90G. This is evident in Figure 4D, as the csHisH90G chevron is shifted downward to slower unfolding kinetics relative to HisH90G. Indeed, csHisH90G has an unfolding rate constant of 3.4 × 10^−3^ s^−1^ at the kinetic midpoint in GuHCl, which is 2.0-fold slower than that of HisH90G (Table 2). So, substituting 3Foil core residues into HisH90G doubles the unfolding half-life, in excellent agreement with the predicted modest effect of these mutations based on LRO, ACO, and C_α_-SBM simulations. Thus, the strategy of using 3Foil core residues to engineer kinetic stability in hisactophilin by increasing long-range intramolecular contacts improved hisactophilin kinetic stability. Significantly, this method of designing protein kinetic stability through the consideration of LRO, ACO, and simulated unfolding free energy barriers provides a rational and predictable route to engineering targeted protein kinetic stability.

### 3.6 csHisH90G folding kinetics suggest a 3Foil-like folding pathway

**FIGURE 5:**
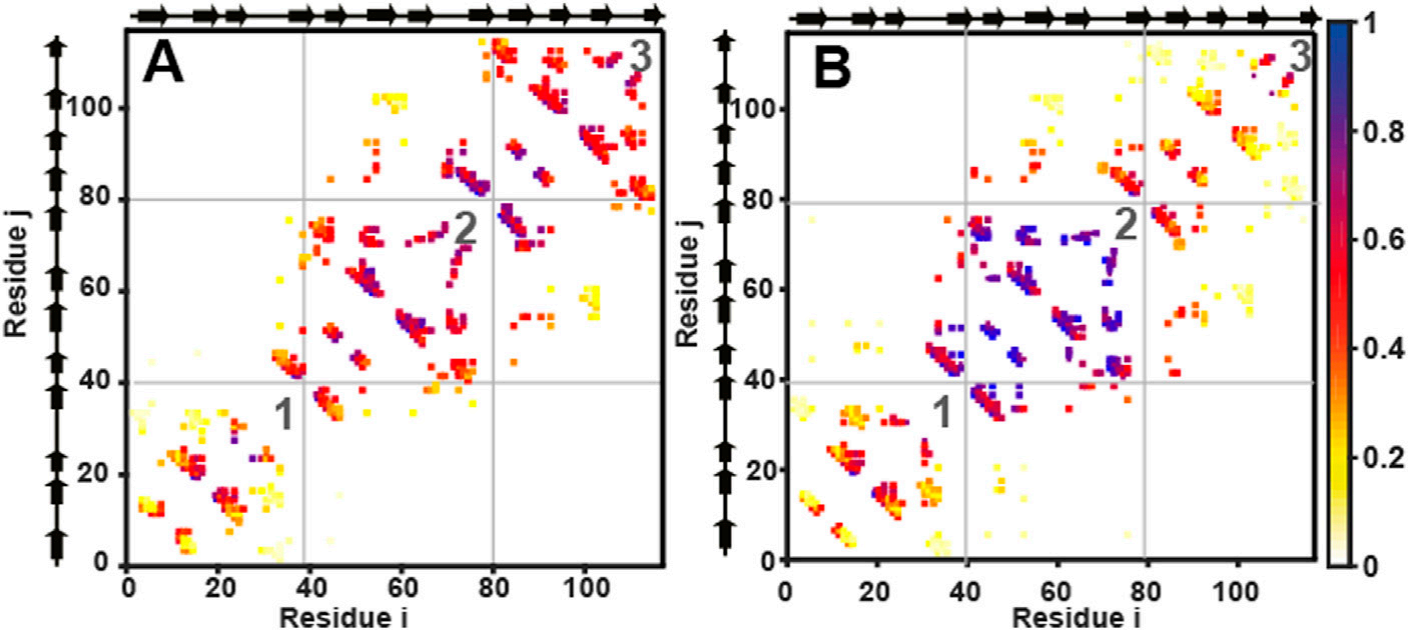
C_α_ structure-based simulations reveal distinct folding pathways for wtHis and csHisH90G. Average contact maps for **(A)** wtHis and **(B)** csHisH90G at Q = 0.4. Average contact maps are a two-dimensional representation of the average partially folded structure of the protein when the protein is 40% folded. Residues are indexed on the x and the *y*-axes. Secondary structural elements of the residues are marked beside the axes on the top and the left. A colored box placed at (*i, j*) and (*j, i*) means that a contact is present between residues *i* and *j*. Contacts are colored based on how formed they are, with one indicating fully formed and 0 indicating unformed. The color bar is given on the right. The N-terminal, central, and C-terminal trefoils are labeled 1, 2, and 3, respectively. Average contact maps for wtHis and csHisH90G show that wtHis initiates folding from its C-terminal trefoil, while folding occurs from the central trefoil in csHisH90G. The average contact map for 3Foil at Q = 0.4 is given in Supplementary Figure S3.

In contrast to HisH90G, the $\beta_{T}$ value for csHisH90G resembles that of 3Foil rather than wtHis (Broom et al., 2015b) (Table 2). csHisH90G and 3Foil have $\beta_{T}$ values of .68 and .66, respectively, whereas wtHis exhibits a $\beta_{T}$ value of .81, indicating that csHisH90G and 3Foil have less native-like transition states than wtHis. C_α_-SBM folding simulations also suggest distinct folding pathways for csHisH90G and wtHis (Figure 5). Folding in csHisH90G simulations initiates from the central trefoil, while folding in wtHis is initiated in its C-terminal trefoil. However, multiple folding pathways are inherent in the landscapes of the quasi-threefold symmetric β-trefoil proteins, making it easy for the protein to switch between dominant folding pathways (Chavez et al., 2006; Gosavi et al., 2006). This effect is likely to be exacerbated in the folding of 3Foil (Supplementary Figure S3), which has perfect threefold symmetry, and therefore we interpret its simulated folding pathway with caution. Hypothesized molecular origins for the differing folding pathways are given in the Supplementary Discussion.

## 4 Discussion

### 4.1 Using simple descriptors of protein topology to engineer kinetic stability

Protein topology or structure, as encoded by the network of interactions or contacts present in the native state, rather than detailed protein energetics, determines how proteins fold. Thus, simple functions of these contact networks, such as LRO and ACO, are able to capture the principal features of protein topology and correlate quantitatively with unfolding free energy barrier heights (Chavez et al., 2004; Broom et al., 2015a). MD simulations of SBMs, which encode these contact networks and ignore attractive non-native interactions, are not only able to model these barrier heights (Chavez et al., 2004; Gosavi et al., 2006; Gosavi, 2013), but can also be used to understand barrier shapes, the population of intermediates, and the folding path (Hills and Brooks, 2009; Noel and Onuchic, 2012; Kmiecik et al., 2016). We used these simple descriptors of protein topology to model and then modulate unfolding barrier heights to directly engineer protein kinetic stability. Specifically, in designing csHisH90G, we aimed to establish engineering long-range intramolecular interactions as a credible strategy for modulating protein kinetic stability and to show that LRO, ACO, and C_α_-SBM unfolding free energy barriers may serve as valuable predictive measures of changes in kinetic stability.

Engineering long-range interaction networks as a method for modulating protein kinetic stability is attractive for several reasons. Previous C_α_-SBM studies of 3Foil show that deleting long-range contacts made by loop residues lowers the 3Foil free energy barrier of unfolding (Broom et al., 2015b), while experimental kinetics from the 3Foil mutant Q71I show that eliminating long-range contacts at key residues decreases kinetic stability (Dubey et al., 2005; Broom et al., 2017). Long-range interactions are encompassed by both LRO and ACO, while short-range contacts are considered directly only by ACO (Gromiha and Selvaraj, 2001; Ivankov et al., 2003). Further, compared to ACO, LRO provides a stronger, more linear correlation with protein unfolding rates for proteins of larger size and variable structure (Broom et al., 2015a). Here, LRO and ACO calculations predicted 4.1-fold and 2.8-fold slower unfolding rate constants at the transition midpoint, respectively, for csHisH90G compared to wtHis (Table 1), which we use as a structural proxy for the pseudo wild type parent protein HisH90G. Experimental folding kinetics show that csHisH90G has 2.0-fold slower unfolding kinetics relative to HisH90G (Table 2; Figure 4D). So, in the case of csHisH90G, ACO may provide a more accurate prediction of the unfolding rate constant than LRO. Additional kinetic stability designs using proteins of variable size and structure must be pursued to investigate whether LRO or ACO is more accurate in predicting protein kinetic stability.

### 4.2 Alternate methods of engineering kinetic stability

Our method for engineering protein kinetic stability is founded on the definition of kinetic stability, i.e., tuning the height of the free energy barrier between the native protein and the transition state. In contrast, previous studies aimed to modulate kinetic stability indirectly by engineering protein characteristics that show experimental correlation with protein kinetic stability. For example, engineering strategies that target disulfide bonds (Mansfeld et al., 1997; Van den Burg et al., 1999; Liu et al., 2021), residues with high B-factors (Le et al., 2012; Chen et al., 2015; Duan et al., 2016; Liu et al., 2021), or residues with high thermal flexibility (Pikkemaat et al., 2002; Xie et al., 2014; Quezada et al., 2018; Liu et al., 2021) all aim to reduce protein mobility and, consequently, protein unfolding. One way to reduce the flexibility of an amino acid is through mutations that increase its intra-protein contacts, and such mutations are likely to be of a similar nature across all methods. However, local rigidity could also be increased by mutations that tune secondary structural propensities (Geiger-Schuller et al., 2018), and such mutations are likely to be complementary to those seen in our method. This method for engineering kinetic stability relies on global structural measures such as LRO and ACO and is unlikely to be able to capture mutations that tune local sequence energetics and promote the formation of specific secondary structural elements or loops, the effect of the H90G mutation on wtHis being one potential example. It should be noted that increasing thermodynamic stability in many proteins will slow protein unfolding and increase kinetic stability in native-like conditions (Abkevich et al., 1995). However, such increases in kinetic stability may not hold when comparisons are made at the mid-point of the transition (i.e., where the change in thermodynamic stability between the native and unfolded states is 0).

### 4.3 Calculating relative barrier heights: Minutiae of the protein structure may not matter

Several forms and flavors of structure-based models exist that encode the protein structure at different levels of coarse-graining and in slightly different ways (Clementi et al., 2000; Hyeon and Thirumalai, 2011; Noel and Onuchic, 2012; Yadahalli et al., 2014; Noel et al., 2016). Similarly, although a canonical method for determining contacts for LRO and ACO calculations exists (Gromiha and Selvaraj, 2001; Ivankov et al., 2003), these measures could be calculated using other contact definitions (Broom et al., 2015b). These differences in the potential energy function or contact calculation are likely to not have a significant effect on relative barrier heights except in proteins where specific functional features affect folding and need to be encoded accurately (Azia and Levy, 2009; Yadahalli and Gosavi, 2016). This is true because overall protein topology, rather than the details of energetics, determines how a protein folds (Bryngelson et al., 1995; Wolynes et al., 1995; Onuchic et al., 1997; Onuchic and Wolynes, 2004). Conversely, a detailed structure of the protein, such as a high-resolution crystal structure, may not be required to predict relative barrier heights from C_α_-SBM simulations, ACO, and LRO. In fact, higher kinetic stability was achieved here in hisactophilin despite the absence of a crystal structure.

Lack of a high-resolution crystal structure is not an uncommon hurdle in protein engineering. Crystal structures currently comprise only ∼100,000 unique proteins of billions of known protein sequences, and known protein sequences outnumber solved protein structures 736 times over (Muhammed and Aki-Yalcin, 2019; Jumper et al., 2021). Recent advances in structure prediction, e.g., AlphaFold2 (Jumper et al., 2021), ColabFold (Mirdita et al., 2022), and RoseTTAFold (Baek et al., 2021), now enable the generation of structural models (which may be suitable for C_α_-SBM simulations and ACO and LRO calculations) with significant confidence from just the primary sequence. Consequently, our method may be applied to the large proportion of proteins that lack high-resolution crystal structures. That being said, a “reasonable” and complete structure is required for C_α_-SBM simulations and LRO and ACO calculations.

### 4.4 Core engineering can be performed without close sequence homologues

Many contemporary protein stability design strategies require the use of close sequence homologues and a multiple sequence alignment (MSA). For example, MSAs are used in consensus design (Broom et al., 2012; Feng et al., 2016; Sternke et al., 2019) and coevolution analysis (Reynolds et al., 2013; Ovchinnikov et al., 2014; Swint-Kruse, 2016). As with high-resolution crystal structures, many proteins lack a sufficient number of homologous sequences to benefit from these strategies. In fact, of the sequences in UniProtKB, 23% match no Pfam entry (Mistry et al., 2021). Despite belonging to the β-trefoil lineage, hisactophilin lacks closely related sequence homologues, precluding application of consensus or coevolution design. However, hisactophilin is an ideal test protein for our method for designing kinetic stability because it has close structural homologues with distinctly different kinetic stabilities. Further, strong conservation of core residue hydrophobicity but not identity across β-trefoil sequences, including wtHis and 3Foil, suggested that the β-trefoil core may tolerate the re-engineering of its interaction network, the basis of our strategy to design kinetic stability.

The results presented here recommend engineering of protein cores as an attractive and accessible target for increasing protein kinetic stability. Namely, swapping entire networks of core interactions between homologous proteins may be widely applicable. Improving protein core packing is generally associated with augmented van der Waals interactions, more favorable core residue side chain steric interactions, and increased burial of hydrophobic surface area, all of which are known to increase protein stability (Ventura and Serrano, 2004; Borgo and Havranek, 2012; Kim et al., 2012). Further, core engineering is among the most well-developed, predictable, and feasible strategies in protein design. Mutagenesis studies show that protein core sequences can be highly amenable to hydrophobic mutations, including complete core redesign, while retaining the protein fold (Kuhlman and Baker, 2000; Ng et al., 2007; Murphy et al., 2012; Ben-David et al., 2019; Koga et al., 2020). Protein design software, including SCWRL (Krivov et al., 2009), OSCAR (Liang et al., 2011), RASP (Miao et al., 2011), Rosetta (Kuhlman and Baker, 2000), SCCOMP (Eyal et al., 2004)], and FoldX (Guerois et al., 2002), achieve higher accuracy for predicting favorable core residue conformations compared to surface residues, offering higher rates of success for *de novo* core mutant designs (Peterson et al., 2014; Gaines et al., 2017; Broom et al., 2020). Core residues are generally highly conserved as hydrophobic (Kuhlman and Baker, 2000; Ben-David et al., 2019), making core engineering amenable to bioinformatic design strategies such as consensus and covarying residue design. Significant to our method for designing kinetic stability, consideration of alternate core packing is well suited to optimizing both van der Waals and steric interactions to increase a protein’s LRO and ACO. Further, since core residue positions tend to be conserved among homologous proteins while specific residue identities can vary, core residues lend themselves as promising targets for designs that aim to swap entire networks of protein interactions, such as that reported here.

Beyond the demonstrated success of engineering core residues, this approach may allow protein stabilization while generally maintaining function. For example, core residue engineering may minimally effect function in β-trefoil proteins, as β-trefoils achieve ligand-binding through surface loop residues (Figure 1) (Brych et al., 2004; Olsen et al., 2004; Gosavi et al., 2008; Broom et al., 2012; Terada et al., 2017; Blaber, 2022) and display considerable plasticity in their core packing arrangements (Murzin et al., 1992; Ponting and Russell, 2000; Longo and Blaber, 2013; Blaber, 2022). So, in the absence of allosteric affects, β-trefoil proteins with desirable functionality but only moderate stability may gain kinetic and thermodynamic stability from the replacement of core residues with those of another β-trefoil or from *de novo* core repacking. While allostery between the surface and the core may modulate binding (Giri Rao and Gosavi, 2015; Ben-David et al., 2019), a variety of scaffolds bind diverse ligands just *via* loops (e.g., antibodies (Warszawski et al., 2019), adnectins/monobodies (Ng et al., 2007; Trainor et al., 2022), and repeat proteins (Hansen et al., 2017)]. Such proteins are attractive targets for engineering increased kinetic stability through increased long-range core contacts. Thus, optimization of core interactions for desired kinetic stability can provide a valuable tool for practical engineering of functional proteins. In addition, developing a scaffold with high kinetic stability may provide a particularly useful starting point for subsequent engineering of function, which often impairs stability (Liu et al., 2001; Gosavi, 2013; Tenorio et al., 2022). Core residue engineering may be even more accessible for proteins containing repetitive structures and symmetry such as the structurally symmetric β-trefoils and TIM barrels, or repeat proteins (Meiering et al., 1991; Sancho et al., 1991; Broom et al., 2015b; Broom et al., 2016; Vrancken et al., 2020). Such proteins are of interest for their multivalent binding of identical or distinct ligands. Finally, stabilizing proteins by engineering core residues may better promote protein crystallization and aid structural characterization of surface binding and catalytic sites.

## 5 Conclusion

Our strategy for predicting and modulating protein kinetic stability is readily applicable to other protein families. Specifically, residues contributing to kinetic stability within a protein fold may be identified by comparing networks of long-range contacts between homologous proteins of differing kinetic stabilities. Self-contained long-range interaction networks, such as those between conserved hydrophobic core residues, may then be swapped between homologous proteins to achieve the targeted kinetic stability. Due to the nature of using structurally homologous proteins, parent proteins need not have closely related primary sequences so long as they share a common fold. Importantly, using LRO, ACO, and C_α_-SBM unfolding free energy barrier predictions, hybrid proteins that fail to reach the desired kinetic stability are identifiable before embarking on experiments. Fundamentally, these predictive measures can be used to evaluate the change in kinetic stability for any multi mutant protein relative to its parent protein. We envision this method may be extended to also incorporate novel long-range interactions, which may also be designed *de novo*. Thus, our method of engineering long-range intramolecular interactions and using protein topology measures and coarse-grained free energy barriers to modulate and predict *in vitro* kinetic stability is widely applicable in the design of hybrid and multi mutant proteins for fundamental and practical purposes.

## Data Availability

The raw data supporting the conclusions of this article will be made available by the authors, without undue reservation.
