# Supplementary material for: Engineering the kinetic stability of a β-trefoil protein by tuning its topological complexity
Source: Front Mol Biosci. 2023 Feb 8;10:1021733. doi: 10.3389/fmolb.2023.1021733 (PMC9945329; doi:10.3389/fmolb.2023.1021733)
Supplement: Supplementary file 1 [file DataSheet1.PDF]

Supplementary Information

Supplementary Methods

Modeling the 3Foil free energy barrier of unfolding

Folding and unfolding transitions were exceedingly rare during 3Foil C_α-SBM simulations owing to 3Foil's free energy barrier of unfolding, which was previously shown to be unusually high (Broom et al., 2015). Since high energy conformations in configuration space could not be sufficiently sampled, a reliable estimation of the 3Foil unfolding free energy barrier could not be obtained using direct sampling. Thus, we implemented the enhanced sampling method replica exchange umbrella sampling (REUS) at an estimated folding temperature (T_f) to model 3Foil's folding trajectory and unfolding free energy barrier (Kästner, 2011; Giri Rao and Gosavi, 2018).

REUS simulations for 3Foil were carried out using GROMACS v.4.5.4 patched with PLUMED v.1.3, which allows enhanced-sampling methods and MPI processing (Bekker, H., Berendsen, H. J. C., Dijkstra, E. J., Achterop, S., van Drunen, R. et al., 1993; Berendsen et al., 1995; Lindahl et al., 2001; Van Der Spoel et al., 2005; Hess et al., 2008; Bonomi et al., 2009). REUS simulations were set up as described previously (Giri Rao and Gosavi, 2018) using 34 replica windows, where each window is centered upon a unique value of Q, the fraction of native contacts. This Q was calculated using a smoothing function as in PLUMED and is also defined in Giri Rao and Gosavi (2018). A production simulation for each of the 34 windows was run for 300 ns ($6x10^8$ steps) with a force constant of 0.05 and replica exchange attempted every 5 ps (10000 steps; 59999 exchange attempts in total). The unbiased free energy profile (FEP) was obtained using the weighted histogram analysis method (Kumar et al., 1992; Gallicchio et al., 2005), the criteria of which are given in Figure S2 A-C. Finally, we modeled 3Foil's unbiased free energy barrier of unfolding at the folding temperature (Figure S2 D), which was in overall agreement with the previous unfolding free energy barrier modeled using a different contact map definition (Broom et al., 2015).

The folding pathway of 3Foil was assessed by examining the average contact maps along the progress coordinate Q. The average contact map represents the average probability of the formation of all native contacts at a given Q value along the folding pathway. As in the FEP of 3Foil, WHAM analysis was first performed to obtain unbiased probabilities of native contacts. The average contact maps are then calculated by averaging the contact probability matrices around a given Q value (± 0.025). Figure S3 shows the average contact map of 3Foil at around the transition state ensemble of $Q \approx 0.4$.

Supplementary Discussion

The central goal of this study was to develop a strategy for the engineering of increased protein kinetic stability. It is important to note that kinetic stability does not, in general, predict thermodynamic stability, because the latter is defined by the ratio of both the unfolding and folding rates. Still, increasing the kinetic stability of a protein by increasing its long-range contacts will slow the unfolding rate and so contribute to increased thermodynamic stability. However, this effect will be offset by concomitant slowing of the folding rate, as is also observed experimentally here (Figure 4D; Table 2) and provides additional support for our design strategy. While thermodynamic stability is also increased with increased kinetic stability in csHisH90G, predicting the change in thermodynamic stability is a distinct challenge because changing the size and stereochemistry of core residues has wide-ranging effects on stability (Shental-Bechor et al., 2012; Nisthal et al., 2019; MacKenzie et al., 2022; Pucci et al., 2022). Below, we consider some additional aspects of the molecular basis of the kinetic and thermodynamic stability pertinent to the present study.

3Foil core residues in hisactophilin modestly increase long-range contacts, topological complexity, and kinetic stability

Though engineering 3Foil core residues into wtHis introduces 74 new contacts in csHisH90G from protein repacking and from larger residues in the core, most new core contacts do not contribute to LRO. According to the definition for LRO (see Methods), long-range contacts occur between residues that are at least 12 residues apart in the primary sequence. However, in wtHis and csHisH90G, no core residues in adjacent β-strands in the hairpin cap or in neighboring trefoils in the β-barrel, with the exception of β-strands 1 and 12, are more than 11 residues apart in the primary sequence. So, bringing core residue side chains of neighboring trefoils into closer proximity by introducing larger side chains does not increase LRO in csHisH90G, and long-range contacts are gained primarily between β-barrel core residues from the same trefoil. This is owing to hisactophilin's relatively short β2-β3 loops and tight hairpin turns, which are longer in other β-trefoil proteins (Murzin et al., 1992; Gosavi, 2013; Terada et al., 2017; Kimura et al., 2020). In 3Foil, adjacent β-strands in the hairpin cap region are preceded by longer β2-β3 loops such that core residues in the hairpins are 13 residues apart in the primary sequence. Additionally, 3Foil's β-barrel includes longer turns between sequential β-strands such that core residues in the B layer are 12 residues apart in the primary sequence for neighboring trefoils (Figure 2). So, due to longer loops, the hairpin cap and B layer core residues in 3Foil may form long-range contacts to all adjacent β-strands, both within and between trefoils. While core residues in csHisH90G and 3Foil likely occupy similar positions in space, as suggested by their strikingly similar fluorescence spectra (Figure 4B) (Broom et al., 2012), 3Foil core residues form 53 long-range core-core contacts and csHisH90G core residues make only 39. Thus, short turns and loops in csHisH90G limit core residue capacity to form long-range contacts that may otherwise result in greater gain in topological complexity, as in 3Foil. Collectively, the increase in long-range contacts by core residues represents a small proportion of the total protein intramolecular contacts (25 new long-range contacts from core residues of 370 total contacts in csHisH90G) and so is expected to result

in a modest (~2-fold) increase in kinetic stability at the unfolding midpoint. Notably, the predicted increase is in close agreement with experiment.

3Foil residues enhance hisactophilin thermodynamic stability

csHisH90G's considerable thermodynamic stabilization was not necessarily expected, given that 3Foil has moderate thermodynamic stability compared to wtHis (Table S1 of Broom et al., (2015)) and the effects of changing the stereochemistry and size of core residues are unpredictable, as noted above. 3Foil core residues increasing hisactophilin thermodynamic stability suggests that the 3Foil core is thermodynamically stable and 3Foil is thermodynamically destabilized by other features. A trade-off between stability and function is a well-known phenomenon, also evident in β-trefoil proteins (Meiering et al., 1991; Sancho et al., 1991; Fersht, 1999; Liu et al., 2001; Broom et al., 2015). As such, 3Foil's functional loops are a likely source of 3Foil's diminished thermodynamic stability (Broom et al., 2015). Similarly, Symfoil contains core residues similar to those of 3Foil and gained hyperthermostability with the concurrent loss of heparin-binding functionality over several iterative rounds of design (Lee and Blaber, 2011; Lee et al., 2011). wtHis core residues also contribute to function by forming a deep pocket that accommodates the hydrophobic acyl chain of a covalently attached N-terminal myristoyl group (Smith et al., 2010; Shental-Bechor et al., 2012; MacKenzie et al., 2022). When the myristoyl group is buried in the wtHis core, the acyl chain makes additional stabilizing interactions with hydrophobic residues, increasing thermodynamic stability (Smith et al., 2010; MacKenzie et al., 2022). Since 3Foil's core residues are larger and more closely packed than those of wtHis, core residues in csHisH90G may accomplish a similar thermodynamically stabilizing effect as the myristoyl group in wtHis. So, wtHis core residues may limit non-myristoylated wtHis thermodynamic stability, and 3Foil core residues in csHisH90G ameliorate this limitation by eliminating the core pocket functionality and facilitating augmented core packing.

Protein thermodynamic stability can also be gained by altering short-ranged contacts; an example is H90G. This mutation introduces a conserved glycine that is found in 3Foil into hisactophilin. Notably, glycine decreases kinetic stability by increasing the unfolding rate at the unfolding midpoint; however, a larger increase in the folding rate causes the thermodynamic stability to increase. These results highlight again the distinctions between kinetic and thermodynamic stability.

Examining the csHisH90G folding pathway

Contact analysis of the wtHis and csHisH90G C-terminal trefoil reveals that several stabilizing interactions present in wtHis are lost in csHisH90G (Figure 3C, D). Specifically, in wtHis, the backbone conformation of E115 is twisted such that the E115 side chain points toward solvent rather than the protein core. Through this unusual conformation, E115 makes long-range, stabilizing charge-charge and charge-polar contacts to residues in the β8-β9 turn. Additionally, the twisted β12 backbone conformation in wtHis also facilitates many interactions between residues

in β1 and β12. In contrast, L115 in csHisH90G points towards the protein core to form hydrophobic contacts with core residues Y4, L36, and L76. This ameliorates the twisted β12 backbone conformation observed in wtHis, but also results in the loss of stabilizing interactions to the β8-β9 turn and between several β1/β12 residues. So, while 3Foil core residues in hisactophilin relieve steric strain in β-strands 1 and 12, concurrent loss of stabilizing interactions from β12 may hinder folding in the C-terminal trefoil. Further experimental investigation of wtHis, csHisH90G, and 3Foil folding pathways is needed to confirm the variable folding observed in folding simulations.

Several studies show that mutating core residues can alter protein folding pathways (Ventura and Serrano, 2004; Dalessio et al., 2005; Wensley et al., 2010; Longo et al., 2014; Blaber, 2022). Given that burial of hydrophobic surface area is widely accepted to drive protein folding (Dill, 1990; Bryngelson et al., 1995; Wolynes et al., 1995; Dill and Maccallum, 2012; Chen et al., 2015), it is unsurprising that 3Foil core residues change the hisactophilin folding pathway. In replacing wtHis core residues with those of 3Foil, we estimate that the hydrophobic surface area of core residues increases from 1750 Å^2 in wtHis to 2240 Å^2 in csHisH90G. Completely symmetric designed proteins (i.e. Symfoil and Phifoil) have been shown to have multiple redundant folding nuclei (Longo et al., 2014; Blaber, 2022; Tenorio et al., 2022). Thus, 3Foil core residues may change the hisactophilin folding pathway by providing an alternate folding nucleus (or nuclei) from which to initiate protein folding.

Supplemental Figures

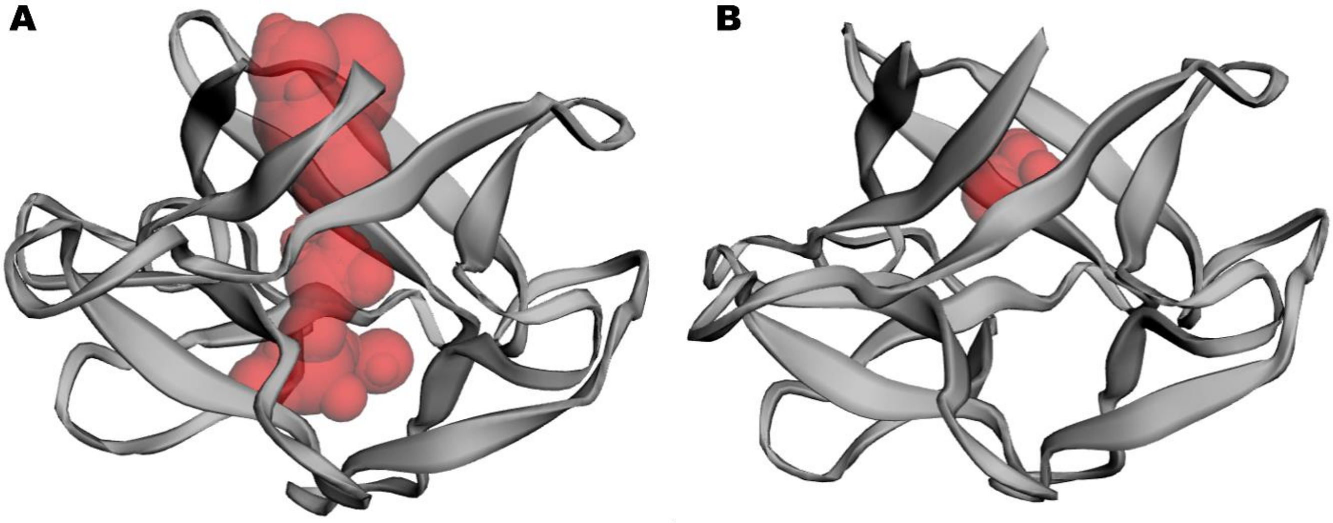

Figure S1. csHisH90G core cavity volume is significantly reduced compared to wtHis. Core cavity volumes were calculated for **(A)** wtHis and **(B)** csHisH90G using Computed Altas of Surface Topography of proteins (CASTp) (Tian et al., 2018). Cavity volumes were calculated using a 1.4 Å radius probe. CASTp identified a 64.8 $Å^3$ cavity that spans the entire wtHis core. csHisH90G exhibits a substantially smaller cavity of 2.57 $Å^3$, indicating that 3Foil core residues significantly reduce empty space in hisactophilin's core. No core cavity was detectable for 3Foil (data not shown).

A

Replica window	0	1	2	3	4	5	6	7	8	9	10	11
Q (10^{-2})	2.5	5.5	8.6	11.6	14.7	17.7	20.8	23.8	26.9	29.9	32.6	35.2
Replica exchange probability (10^{-2})	41	32	28	26	25	24	23	22	22	26	24	

Replica window	11	12	13	14	15	16	17	18	19	20	21	22
Q (10^{-2})	35.2	37.8	40.5	43.1	45.8	48.4	51.1	53.7	56.3	59.0	61.6	64.3
Replica exchange probability (10^{-2})	24	26	28	30	30	28	25	26	25	25	25	

Replica window	22	23	24	25	26	27	28	29	30	31	32	33
Q (10^{-2})	64.3	66.9	69.5	72.6	75.6	78.7	81.7	84.8	87.8	90.9	93.9	96.9
Replica exchange probability (10^{-2})	26	28	23	24	26	28	30	32	34	37	41	

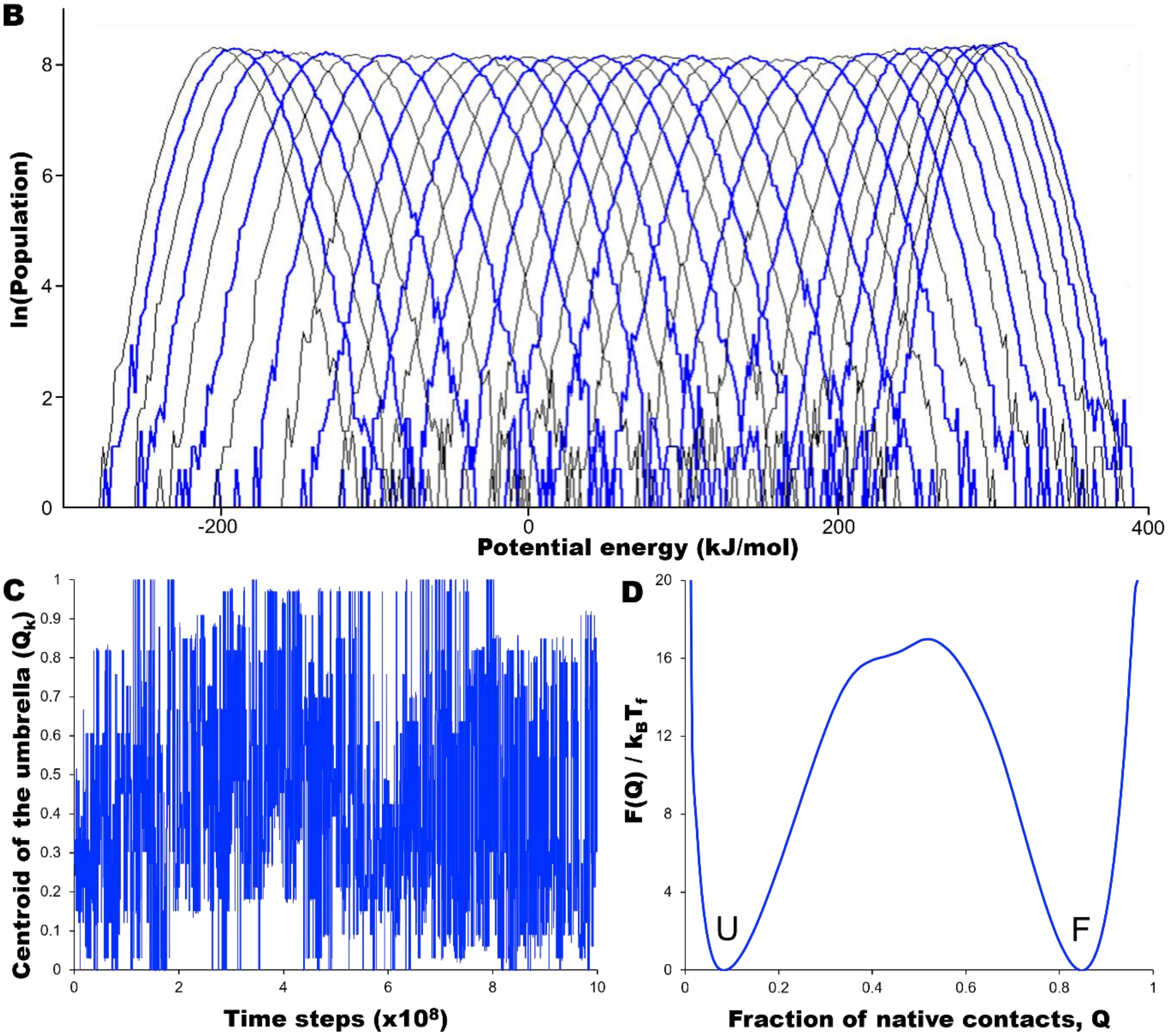

Figure S2. 3Foil REUS simulations using 34 windows meet WHAM criteria. 3Foil REUS simulations show that 34 non-uniformly spaced replica windows and a force constant of 0.05 result in good replica exchange statistics and window overlap for WHAM. **(A)** Following REUS, the probability of successful replica exchange between adjacent windows ranges from 0.22 to 0.41 for all windows, in good agreement with the recommended value of 0.2 to 0.4. Replica exchange was attempted every 5 ps (10000 steps), and 59999 replica exchanges were attempted in total. **(B)** The potential energy distribution of a given replica window overlaps with the potential energy distribution of adjacent replica windows for all windows. Odd replica windows are colored black and even replica windows are colored blue to better display the overlap of neighboring windows. Potential energy trajectories were generated using the GROMACS g_energy command. Replica exchange traces and potential energy distributions were visualized in xmgrace (Turner, 2005). **(C)** Tracing exchange events for a given replica (*e.g.* replica 10 (above)) shows that each replica exchanged into all other replica windows, where the exchange number increases with time during the simulation. **(D)** 3Foil unfolding free energy barrier modeled from unbiased REUS simulation data solved at T = 160.1 K using WHAM (Gallicchio et al., 2005). The unfolding free energy barrier is along the progress coordinate Q, the fraction of native contacts. The unfolded (U) and folded (F) states are indicated. Unfolding free energy barrier heights are given in Table 1. It should be noted that simulation temperatures cannot be directly compared to experimental temperatures in coarse-grained simulations.

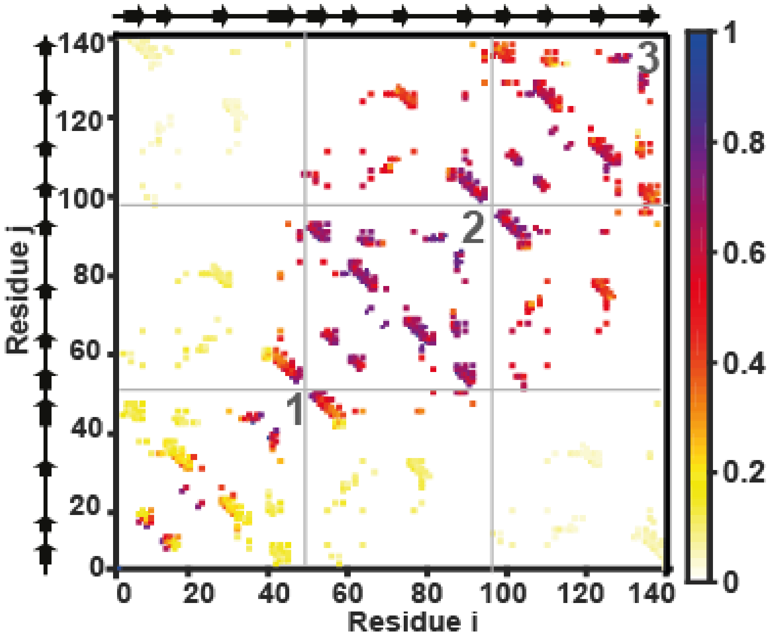

Figure S3. Average contact map for 3Foil at Q = 0.4 at around the transition state ensemble. Contacts are colored based on degree of structure, with 1 indicating native levels of structure and 0 indicating random coil. The N-terminal, central, and C-terminal trefoil are labeled 1, 2, and 3, respectively. 3Foil initiates early folding from the central trefoil and then continues with the C-terminal and central trefoils together, as can be seen here. Transition state ensembles for wtHis and csHisH90G are given in Figure 5.

Contact lists

wtHis

(2, 34), (2, 35), (2, 36), (2, 117), (3, 33), (3, 34), (3, 35), (3, 116), (3, 117), (3, 118), (4, 13), (4, 14), (4, 15), (4, 30), (4, 31), (4, 32), (4, 33), (4, 34), (4, 116), (4, 117), (4, 118), (5, 13), (5, 14), (5, 15), (5, 34), (5, 114), (5, 115), (5, 116), (5, 117), (6, 12), (6, 13), (6, 14), (6, 36), (6, 45), (6, 76), (6, 83), (6, 85), (6, 113), (6, 114), (6, 115), (6, 116), (7, 12), (7, 13), (7, 112), (7, 113), (7, 114), (7, 116), (8, 12), (8, 13), (8, 14), (8, 23), (8, 95), (8, 97), (8, 112), (8, 113), (9, 97), (9, 112), (9, 113), (9, 114), (10, 25), (10, 114), (11, 24), (11, 25), (11, 97), (12, 23), (12, 24), (12, 25), (12, 95), (12, 97), (13, 22), (13, 23), (13, 24), (13, 25), (13, 26), (13, 31), (13, 116), (14, 22), (14, 23), (14, 34), (14, 53), (14, 85), (14, 93), (14, 94), (14, 95), (14, 101), (14, 113), (15, 20), (15, 21), (15, 22), (15, 23), (15, 24), (15, 31), (15, 32), (15, 33), (15, 34), (16, 20), (16, 21), (16, 22), (16, 24), (16, 32), (16, 33), (16, 34), (16, 47), (17, 21), (17, 22), (17, 24), (17, 28), (17, 30), (17, 32), (18, 32), (21, 34), (21, 53), (21, 63), (22, 34), (23, 101), (24, 28), (24, 30), (24, 31), (24, 32), (25, 31), (26, 31), (31, 116), (33, 46), (33, 47), (33, 118), (34, 45), (34, 46), (34, 47), (34, 53), (34, 63), (35, 44), (35, 45), (35, 46), (35, 47), (36, 43), (36, 44), (36, 45), (36, 76), (37, 42), (37, 43), (37, 44), (37, 46), (38, 42), (38, 43), (39, 43), (39, 44), (39, 52), (39, 66), (39, 67), (39, 68), (39, 73), (40, 68), (41, 75), (42, 69), (42, 70), (42, 73), (42, 74), (42, 75), (43, 73), (43, 74), (43, 75), (43, 76), (44, 52), (44, 67), (44, 72), (44, 73), (44, 74), (45, 51), (45, 52), (45, 53), (45, 72), (45, 73), (45, 74), (45, 76), (45, 85), (46, 50), (46, 51), (46, 52), (47, 51), (49, 65), (50, 64), (50, 65), (51, 63), (51, 64), (52, 62), (52, 63), (52, 64), (52, 65), (52, 66), (52, 67), (52, 72), (52, 73), (53, 61), (53, 62), (53, 63), (53, 64), (53, 65), (53, 71), (53, 72), (53, 74), (53, 85), (53, 93), (54, 60), (54, 61), (54, 62), (54, 63), (54, 64), (54, 65), (54, 71), (54, 72), (54, 74), (55, 60), (55, 61), (55, 62), (55, 71), (55, 74), (55, 87), (55, 88), (55, 91), (55, 93), (55, 102), (55, 103), (56, 60), (56, 71), (57, 103), (58, 102), (58, 103), (59, 100), (59, 101), (59, 102), (59, 103), (60, 103), (61, 93), (61, 101), (61, 102), (61, 103), (62, 71), (64, 72), (65, 72), (67, 72), (67, 73), (68, 73), (69, 73), (70, 74), (70, 75), (70, 86), (70, 87), (70, 88), (71, 86), (71, 87), (71, 88), (71, 89), (71, 91), (73, 86), (73, 87), (74, 85), (74, 86), (74, 87), (74, 91), (74, 93), (75, 84), (75, 85), (75, 86), (76, 83), (76, 84), (76, 85), (76, 86), (77, 82), (77, 83), (77, 84), (78, 82), (78, 83), (78, 84), (78, 115), (79, 83), (79, 84), (79, 107), (79, 108), (79, 112), (81, 113), (81, 114), (81, 115), (82, 108), (82, 109), (82, 112), (82, 113), (82, 114), (82, 115), (83, 111), (83, 112), (83, 113), (83, 114), (83, 115), (84, 92), (84, 107), (84, 111), (84, 112), (84, 113), (85, 91), (85, 92), (85, 93), (85, 111), (85, 113), (86, 90), (86, 91), (86, 92), (87, 91), (89, 105), (90, 104), (90, 105), (91, 103), (91, 104), (91, 105), (92, 102), (92, 103), (92, 104), (92, 105), (92, 106), (92, 107), (92, 111), (93, 101), (93, 102), (93, 103), (93, 110), (93, 111), (93, 113), (94, 100), (94, 101), (94, 102), (94, 103), (94, 104), (94, 110), (94, 111), (95, 100), (95, 101), (95, 109), (95, 110), (95, 111), (95, 113), (96, 100), (96, 102), (96, 104), (96, 110), (101, 113), (103, 111), (104, 110), (104, 111), (106, 110), (106, 111), (107, 111), (107, 112), (108, 112)

Total contacts: 352

csHisH90G

(2, 34), (2, 35), (2, 36), (2, 117), (2, 118), (3, 33), (3, 34), (3, 35), (3, 116), (3, 117), (3, 118), (4, 14), (4, 15), (4, 32), (4, 33), (4, 34), (4, 35), (4, 36), (4, 115), (4, 116), (4, 117), (4, 118), (5, 13), (5, 14), (5, 15), (5, 33), (5, 34), (5, 114), (5, 115), (5, 116), (5, 118), (6, 12), (6, 13), (6, 14), (6, 34), (6, 45), (6, 53), (6, 76), (6, 83), (6, 85), (6, 93), (6, 113), (6, 114), (6, 115), (7, 11), (7, 12), (7, 13), (7, 112), (7, 113), (7, 114), (7, 116), (8, 12), (8, 95), (8, 112), (9, 95), (9, 97), (9, 109), (9, 110), (9, 112), (10, 25), (10, 95), (10, 97), (10, 98), (11, 24), (11, 25), (12, 23), (12, 24), (12, 25),

(12, 95), (12, 97), (12, 98), (12, 113), (13, 22), (13, 23), (13, 24), (13, 25), (13, 26), (13, 31), (13, 33), (13, 116), (14, 21), (14, 22), (14, 23), (14, 24), (14, 33), (14, 34), (14, 53), (14, 93), (14, 100), (14, 101), (14, 113), (15, 20), (15, 21), (15, 22), (15, 24), (15, 31), (15, 32), (15, 33), (15, 34), (16, 20), (16, 21), (16, 22), (16, 24), (16, 31), (16, 32), (16, 33), (16, 34), (16, 47), (16, 48), (17, 22), (17, 24), (17, 30), (17, 31), (17, 32), (18, 32), (18, 48), (21, 34), (21, 47), (21, 53), (21, 63), (21, 100), (22, 31), (22, 100), (23, 97), (23, 100), (23, 101), (24, 31), (24, 32), (25, 31), (26, 31), (27, 31), (32, 47), (32, 48), (33, 46), (33, 47), (33, 116), (33, 118), (34, 45), (34, 46), (34, 47), (34, 51), (34, 52), (34, 53), (34, 63), (35, 44), (35, 45), (35, 46), (35, 47), (36, 43), (36, 44), (36, 45), (36, 76), (36, 115), (37, 42), (37, 43), (37, 44), (37, 46), (37, 52), (38, 42), (38, 43), (39, 43), (39, 44), (39, 46), (39, 52), (39, 66), (39, 67), (39, 68), (39, 73), (41, 75), (42, 69), (42, 70), (42, 73), (42, 74), (42, 75), (42, 76), (42, 86), (42, 87), (43, 72), (43, 73), (43, 74), (43, 75), (43, 76), (44, 52), (44, 66), (44, 72), (44, 73), (44, 74), (45, 51), (45, 52), (45, 53), (45, 63), (45, 72), (45, 73), (45, 74), (45, 76), (45, 85), (46, 50), (46, 51), (46, 52), (46, 63), (47, 51), (47, 63), (50, 64), (50, 65), (50, 66), (51, 63), (51, 64), (52, 62), (52, 63), (52, 64), (52, 65), (52, 66), (52, 72), (52, 73), (53, 61), (53, 62), (53, 63), (53, 71), (53, 72), (53, 73), (53, 74), (53, 85), (53, 93), (54, 60), (54, 61), (54, 62), (54, 63), (54, 64), (54, 65), (54, 71), (54, 72), (54, 74), (54, 93), (55, 60), (55, 61), (55, 62), (55, 71), (55, 74), (55, 87), (55, 88), (55, 91), (55, 93), (55, 103), (56, 60), (56, 71), (56, 88), (56, 103), (57, 91), (58, 102), (58, 103), (59, 96), (59, 101), (59, 102), (59, 103), (60, 102), (60, 103), (61, 93), (61, 100), (61, 101), (61, 103), (62, 71), (62, 72), (64, 72), (65, 72), (66, 72), (66, 73), (67, 71), (67, 72), (67, 73), (68, 73), (69, 73), (70, 87), (70, 88), (71, 87), (71, 88), (73, 86), (73, 87), (74, 85), (74, 86), (74, 87), (74, 91), (74, 93), (74, 103), (75, 84), (75, 85), (75, 86), (76, 83), (76, 84), (76, 85), (76, 115), (77, 82), (77, 83), (77, 84), (77, 86), (77, 92), (77, 107), (78, 82), (78, 83), (79, 84), (79, 92), (79, 107), (79, 112), (81, 113), (81, 114), (82, 112), (82, 113), (82, 114), (83, 111), (83, 112), (83, 113), (83, 114), (83, 115), (84, 92), (84, 93), (84, 107), (84, 111), (84, 112), (84, 113), (85, 91), (85, 92), (85, 93), (85, 111), (85, 113), (86, 90), (86, 91), (86, 92), (87, 91), (87, 93), (90, 104), (90, 105), (91, 103), (91, 104), (91, 105), (92, 102), (92, 103), (92, 104), (92, 106), (92, 107), (92, 111), (93, 101), (93, 102), (93, 103), (93, 110), (93, 111), (93, 113), (94, 100), (94, 101), (94, 102), (94, 103), (94, 104), (94, 110), (94, 111), (95, 100), (95, 101), (95, 110), (95, 112), (95, 113), (96, 100), (96, 102), (96, 104), (96, 110), (97, 110), (101, 113), (104, 108), (104, 110), (104, 111), (106, 111), (107, 111), (107, 112), (108, 112)

Total contacts: 370

3Foil

(2, 141), (2, 142), (3, 42), (3, 58), (3, 141), (3, 142), (4, 43), (4, 44), (4, 45), (4, 141), (4, 142), (5, 42), (5, 43), (5, 44), (5, 45), (5, 56), (5, 58), (5, 140), (5, 141), (5, 142), (6, 41), (6, 42), (6, 43), (6, 44), (6, 45), (6, 139), (6, 140), (6, 141), (6, 142), (7, 14), (7, 16), (7, 35), (7, 41), (7, 42), (7, 43), (7, 138), (7, 139), (7, 140), (7, 142), (8, 15), (8, 16), (8, 17), (8, 41), (8, 43), (8, 45), (8, 55), (8, 64), (8, 102), (8, 111), (8, 137), (8, 138), (8, 139), (8, 140), (9, 13), (9, 14), (9, 15), (9, 16), (9, 35), (9, 99), (9, 136), (9, 137), (9, 138), (9, 140), (10, 14), (10, 15), (10, 17), (10, 32), (10, 113), (10, 133), (10, 134), (10, 136), (10, 137), (10, 138), (11, 97), (11, 99), (11, 133), (11, 134), (11, 136), (11, 138), (12, 32), (12, 113), (12, 114), (12, 116), (12, 117), (12, 118), (12, 133), (12, 134), (12, 136), (13, 32), (13, 117), (13, 118), (14, 34), (14, 35), (14, 138), (15, 32), (15, 33), (15, 34), (15, 35), (15, 118), (15, 120), (15, 137), (16, 31), (16, 32), (16, 33), (16, 35), (16, 41), (17, 30), (17, 31), (17, 40), (17, 41), (17, 43), (17, 55), (17, 64), (17, 77), (17, 102), (17, 111), (17, 124), (17, 137), (18, 22), (18, 29), (18, 30), (18, 31), (18, 32), (18, 33), (18, 40), (18, 41), (19, 23), (19, 28), (19, 29), (19, 30), (19, 40), (19, 43), (19, 57), (19, 59), (19, 79), (20, 28), (20, 29), (20, 30), (20,

31), (20, 121), (21, 31), (21, 40), (22, 40), (22, 59), (23, 28), (23, 59), (23, 60), (23, 79), (24, 59), (24, 60), (24, 62), (24, 79), (25, 62), (25, 79), (26, 62), (26, 79), (26, 80), (26, 81), (27, 67), (27, 78), (27, 79), (27, 80), (28, 77), (28, 78), (28, 79), (29, 76), (29, 77), (29, 78), (29, 123), (29, 124), (30, 43), (30, 64), (30, 76), (30, 77), (30, 78), (30, 79), (30, 111), (30, 122), (30, 123), (30, 124), (31, 121), (31, 122), (31, 123), (31, 124), (31, 137), (32, 113), (32, 117), (32, 118), (32, 119), (32, 120), (32, 121), (32, 122), (32, 123), (32, 124), (32, 137), (33, 41), (33, 120), (33, 121), (34, 41), (34, 120), (35, 41), (35, 42), (35, 142), (36, 40), (36, 41), (37, 41), (37, 42), (38, 42), (39, 57), (39, 58), (39, 59), (40, 57), (40, 58), (40, 59), (41, 142), (42, 56), (42, 57), (42, 58), (42, 59), (42, 142), (43, 55), (43, 56), (43, 57), (43, 62), (43, 64), (43, 78), (43, 79), (43, 142), (44, 54), (44, 55), (44, 56), (44, 58), (45, 53), (45, 54), (45, 55), (45, 92), (45, 139), (46, 52), (46, 53), (46, 54), (46, 55), (46, 56), (47, 51), (47, 52), (47, 53), (48, 52), (48, 53), (48, 54), (48, 82), (48, 88), (48, 89), (48, 90), (49, 105), (50, 91), (50, 105), (51, 90), (51, 91), (51, 92), (52, 89), (52, 90), (52, 91), (52, 92), (52, 103), (52, 104), (52, 105), (53, 88), (53, 89), (53, 90), (53, 91), (53, 92), (54, 63), (54, 82), (54, 88), (54, 89), (54, 90), (55, 62), (55, 63), (55, 64), (55, 88), (55, 90), (55, 92), (55, 102), (55, 111), (56, 61), (56, 62), (56, 63), (57, 61), (57, 62), (57, 64), (57, 79), (59, 79), (60, 79), (61, 80), (61, 81), (61, 82), (62, 79), (62, 80), (62, 81), (62, 82), (63, 78), (63, 79), (63, 80), (63, 82), (63, 88), (64, 77), (64, 78), (64, 79), (64, 87), (64, 88), (64, 90), (64, 102), (64, 111), (64, 124), (65, 69), (65, 76), (65, 77), (65, 78), (65, 79), (65, 80), (65, 87), (65, 88), (66, 70), (66, 75), (66, 76), (66, 77), (66, 87), (66, 88), (66, 90), (66, 104), (66, 106), (66, 126), (67, 75), (67, 76), (67, 77), (67, 78), (68, 87), (69, 87), (69, 106), (70, 75), (70, 106), (70, 107), (70, 126), (71, 106), (71, 107), (71, 109), (71, 126), (72, 109), (72, 126), (73, 109), (73, 126), (73, 127), (73, 128), (74, 125), (74, 126), (74, 127), (75, 124), (75, 125), (75, 126), (76, 123), (76, 124), (76, 125), (77, 90), (77, 111), (77, 123), (77, 124), (77, 125), (77, 126), (80, 88), (81, 88), (82, 88), (82, 89), (83, 88), (84, 88), (84, 89), (85, 89), (86, 104), (86, 105), (86, 106), (87, 104), (87, 105), (87, 106), (89, 103), (89, 104), (89, 105), (89, 106), (90, 102), (90, 103), (90, 104), (90, 109), (90, 111), (90, 125), (90, 126), (91, 101), (91, 102), (91, 103), (91, 104), (91, 105), (91, 108), (92, 100), (92, 101), (92, 102), (92, 139), (93, 99), (93, 100), (93, 101), (93, 103), (94, 98), (94, 99), (94, 100), (95, 99), (95, 100), (95, 101), (95, 129), (95, 135), (95, 136), (95, 137), (97, 136), (98, 137), (98, 138), (98, 139), (99, 136), (99, 137), (99, 138), (100, 129), (100, 135), (100, 136), (100, 137), (100, 138), (100, 139), (101, 110), (101, 129), (101, 135), (101, 136), (101, 137), (102, 109), (102, 110), (102, 111), (102, 135), (102, 137), (102, 139), (103, 108), (103, 109), (103, 110), (103, 129), (104, 108), (104, 109), (104, 126), (106, 126), (107, 126), (108, 127), (108, 128), (108, 129), (109, 126), (109, 127), (109, 128), (109, 129), (110, 125), (110, 126), (110, 127), (110, 129), (110, 135), (111, 124), (111, 125), (111, 126), (111, 134), (111, 135), (111, 137), (112, 116), (112, 123), (112, 124), (112, 125), (112, 126), (112, 127), (112, 134), (112, 135), (113, 117), (113, 122), (113, 123), (113, 124), (113, 134), (113, 135), (113, 137), (114, 122), (114, 123), (114, 124), (114, 125), (115, 134), (116, 134), (117, 122), (124, 137), (127, 135), (128, 135), (129, 135), (129, 136), (130, 135), (131, 135), (131, 136), (132, 136)

Total contacts: 498

References

Bekker, H., Berendsen, H. J. C., Dijkstra, E. J., Achterop, S., van Drunen, R., van der, Spoel, D., Sijbers, A., Keegstra, H., Reitsma, B., Renardus, M. K. R., Bekker, H., Berendsen, H., Dijkstra, E., Achterop, S., et al. (1993). Gromacs: A parallel computer for molecular dynamics simulations. *Phys. Comput. 92* 92, 252–256.

Berendsen, H. J. C., van der Spoel, D., and van Drunen, R. (1995). GROMACS: A message-passing parallel molecular dynamics implementation. *Comput. Phys. Commun.* 91, 43–56. doi: 10.1016/0010-4655(95)00042-E.

Blaber, M. (2022). Variable and Conserved Regions of Secondary Structure in the β-Trefoil Fold: Structure Versus Function. *Front. Mol. Biosci.* 9, 1–11. doi: 10.3389/fmolb.2022.889943.

Bonomi, M., Branduardi, D., Bussi, G., Camilloni, C., Provasi, D., Raiteri, P., et al. (2009). PLUMED: A portable plugin for free-energy calculations with molecular dynamics. *Comput. Phys. Commun.* 180, 1961–1972. doi: https://doi.org/10.1016/j.cpc.2009.05.011.

Broom, A., Doxey, A. C., Lobsanov, Y. D., Berthin, L. G., Rose, D. R., Howell, P. L., et al. (2012). Modular evolution and the origins of symmetry: Reconstruction of a three-fold symmetric globular protein. *Structure* 20, 161–171. doi: 10.1016/j.str.2011.10.021.

Broom, A., Ma, S. M., Xia, K., Rafalia, H., Trainor, K., Colón, W., et al. (2015). Designed protein reveals structural determinants of extreme kinetic stability. *Proc. Natl. Acad. Sci. U. S. A.* 112, 14605–14610. doi: 10.1073/pnas.1510748112.

Bryngelson, J. D., Onuchic, J. N., Socci, N. D., and Wolynes, P. G. (1995). Funnels, pathways, and the energy landscape of protein folding: A synthesis. *Proteins Struct. Funct. Bioinforma.* doi: 10.1002/prot.340210302.

Chen, T., Song, J., and Chan, H. S. (2015). Theoretical perspectives on nonnative interactions and intrinsic disorder in protein folding and binding. *Curr. Opin. Struct. Biol.* 30, 32–42. doi: 10.1016/j.sbi.2014.12.002.

Dalessio, P. M., Boyer, J. A., McGettigan, J. L., and Ropson, I. J. (2005). Swapping core residues in homologous proteins swaps folding mechanism. *Biochemistry* 44, 3082–3090. doi: 10.1021/bi048125u.

Dill, K. A. (1990). Dominant forces in protein folding. *Biochemistry* 29, 7133–7155. doi: 10.1021/bi00483a001.

Dill, K. A., and Maccallum, J. L. (2012). The Protein-Folding Problem , 50 Years On. *Science (80-.).* 338, 1042–1047.

Fersht, A. (1999). *Structure and Mechanism in Protein Science: A guide to Enzyme Catalysis and Protein Folding.* 2nd ed. W.H. Freeman.

Gallicchio, E., Andrec, M., Felts, A. K., and Levy, R. M. (2005). Temperature weighted histogram analysis method, replica exchange, and transition paths. *J. Phys. Chem. B* 109, 6722–6731. doi: 10.1021/jp045294f.

Giri Rao, V. V. H., and Gosavi, S. (2018). On the folding of a structurally complex protein to its metastable active state. *Proc. Natl. Acad. Sci. U. S. A.* 115, 1998–2003. doi: 10.1073/pnas.1708173115.

Gosavi, S. (2013). Understanding the Folding-Function Tradeoff in Proteins. *PLoS One* 8. doi: 10.1371/journal.pone.0061222.

Hess, B., Kutzner, C., van der Spoel, D., and Lindahl, E. (2008). GROMACS 4: Algorithms for Highly Efficient, Load-Balanced, and Scalable Molecular Simulation. *J. Chem. Theory Comput.* 4, 435–447. doi: 10.1021/ct700301q.

Kästner, J. (2011). Umbrella sampling. *Wiley Interdiscip. Rev. Comput. Mol. Sci.* 1, 932–942. doi: 10.1002/wcms.66.

Kimura, R., Aumpuchin, P., Hamaue, S., Shimomura, T., and Kikuchi, T. (2020). Analyses of the folding sites of irregular β-trefoil fold proteins through sequence-based techniques and Gō-model simulations. *BMC Mol. Cell Biol.* 21, 1–17. doi: 10.1186/s12860-020-00271-4.

Kumar, S., Rosenberg, J. M., Bouzida, D., Swendsen, R. H., and Kollman, P. A. (1992). THE weighted histogram analysis method for free-energy calculations on biomolecules. I. The method. *J. Comput. Chem.* 13, 1011–1021. doi: 10.1002/jcc.540130812.

Lee, J., and Blaber, M. (2011). Experimental support for the evolution of symmetric protein architecture from a simple peptide motif. *Proc. Natl. Acad. Sci. U. S. A.* 108, 126–130. doi: 10.1073/pnas.1015032108.

Lee, J., Blaber, S. I., Dubey, V. K., and Blaber, M. (2011). A polypeptide "building block" for the β-trefoil fold identified by "top-down symmetric deconstruction." *J. Mol. Biol.* 407, 744–763. doi: 10.1016/j.jmb.2011.02.002.

Lindahl, E., Hess, B., and van der Spoel, D. (2001). GROMACS 3.0: a package for molecular simulation and trajectory analysis. *Mol. Model. Annu.* 7, 306–317. doi: 10.1007/s008940100045.

Liu, C., Chu, D., Wideman, R. D., Houliston, R. S., Wong, H. J., and Meiering, E. M. (2001). Thermodynamics of denaturation of hisactophilin, a β-trefoil protein. *Biochemistry* 40, 3817–3827. doi: 10.1021/bi002609i.

Longo, L. M., Kumru, O. S., Middaugh, C. R., and Blaber, M. (2014). Evolution and design of protein structure by folding nucleus symmetric expansion. *Structure* 22, 1377–1384. doi: 10.1016/j.str.2014.08.008.

MacKenzie, D. W. S., Schaefer, A., Steckner, J., Leo, C. A., Naser, D., Artikis, E., et al. (2022). A fine balance of hydrophobic-electrostatic communication pathways in a pH-switching

protein. *Proc. Natl. Acad. Sci.* 119, e2119686119. doi: 10.1073/pnas.2119686119/-/DCSupplemental.60.

Meiering, E. M., Bycroft, M., and Fersht, A. R. (1991). Characterization of Phosphate Binding in the Active Site of Barnase by Site-Directed Mutagenesis and NMR. *Biochemistry* 30, 11348–11356. doi: 10.1021/bi00111a022.

Murzin, A. G., Lesk, M., and Chothiap, C. (1992). β-Trefoil Fold: Patterns of Structure and Sequence in the Kunitz Inhibitors Interleukin-1β and 1α and Fibroblast Growth Factors. *J. Mol. Biol* 223, 531–543.

Nisthal, A., Wang, C. Y., Ary, M. L., and Mayo, S. L. (2019). Protein stability engineering insights revealed by domain-wide comprehensive mutagenesis. *Proc. Natl. Acad. Sci. U. S. A.* 116, 16367–16377. doi: 10.1073/pnas.1903888116.

Pucci, F., Schwersensky, M., and Rooman, M. (2022). Artificial intelligence challenges for predicting the impact of mutations on protein stability. *Curr. Opin. Struct. Biol.* 72, 161–168. doi: 10.1016/j.sbi.2021.11.001.

Sancho, J., Meiering, E. M., and Fersht, A. R. (1991). Mapping transition states of protein unfolding by protein engineering of ligand-binding sites. *J. Mol. Biol.* 221, 1007–1014. doi: 10.1016/0022-2836(91)80188-Z.

Shental-Bechor, D., Smith, M. T. J., MacKenzie, D., Broom, A., Marcovitz, A., Ghashut, F., et al. (2012). Nonnative interactions regulate folding and switching of myristoylated protein. *Proc. Natl. Acad. Sci. U. S. A.* 109, 17839–17844. doi: 10.1073/pnas.1201803109.

Smith, M. T. J., Meissner, J., Esmonde, S., Wong, H. J., and Meiering, E. M. (2010). Energetics and mechanisms of folding and flipping the myristoyl switch in the β-trefoil protein, hisactophilin. *Proc. Natl. Acad. Sci. U. S. A.* 107, 20952–20957. doi: 10.1073/pnas.1008026107.

Tenorio, C. A., Parker, J. B., and Blaber, M. (2022). Functionalization of a symmetric protein scaffold: Redundant folding nuclei and alternative oligomeric folding pathways. *Protein Sci.* 31, 1–14. doi: 10.1002/pro.4301.

Terada, D., Voet, A. R. D., Noguchi, H., Kamata, K., Ohki, M., Addy, C., et al. (2017). Computational design of a symmetrical β-trefoil lectin with cancer cell binding activity. *Sci. Rep.* 7, 5943. doi: 10.1038/s41598-017-06332-7.

Tian, W., Chen, C., Lei, X., Zhao, J., and Liang, J. (2018). CASTp 3.0: Computed atlas of surface topography of proteins. *Nucleic Acids Res.* 46, W363–W367. doi: 10.1093/nar/gky473.

Turner, P. J. (2005). XMGRACE, Version 5.1.19.

Van Der Spoel, D., Lindahl, E., Hess, B., Groenhof, G., Mark, A. E., and Berendsen, H. J. C. (2005). GROMACS: Fast, flexible, and free. *J. Comput. Chem.* 26, 1701–1718. doi:

10.1002/jcc.20291.

Ventura, S., and Serrano, L. (2004). Designing proteins from the inside out. *Proteins Struct. Funct. Genet.* 56, 1–10. doi: 10.1002/prot.20142.

Wensley, B. G., Batey, S., Bone, F. A. C., Chan, Z. M., Tumelty, N. R., Steward, A., et al. (2010). Experimental evidence for a frustrated energy landscape in a three-helix-bundle protein family. *Nature* 463, 685–688. doi: 10.1038/nature08743.

Wolynes, P. G., Onuchic, J. N., and Thirumalai, D. (1995). Navigating the folding routes. *Science (80-.).* 267, 1619 LP – 1620. doi: 10.1126/science.7886447.
